# Evaluation of natural products bioactivity in cardiovascular diseases utilizing zebrafish models: a comprehensive review

**DOI:** 10.3389/fphar.2025.1713417

**Published:** 2025-11-27

**Authors:** Yiming Wang, Xiang Meng, Xuting Song, Lulu Wang, Xianghe Meng, Xiaoya Du, Ziyoviddin Yusupov, Komiljon Tojibaev, Yunpeng Wang, Min He, Mengmeng Sun

**Affiliations:** 1 Changchun University of Chinese Medicine, Changchun, China; 2 The Jilin Province School-Enterprise Cooperation Technology Innovation Laboratory of Herbal Efficacy Evaluation Based on Zebrafish Model Organisms, Changchun University of Chinese Medicine, Changchun, China; 3 Jilin Provincial International Cooperation Key Laboratory of Traditional Medicine for Prevention and Treatment of Metabolic Diseases, Changchun University of Chinese Medicine, Changchun, China; 4 Wish Technology, Changchun, China; 5 Center for Food Evaluation, State Administration for Market Regulation, Beijing, China; 6 Institute of Botany, Academy of Sciences of Uzbekistan, Tashkent, Uzbekistan; 7 Institute of Agricultural Biotechnology, Jilin Academy of Agricultural Sciences (Northeast Agricultural Research Center of China), Changchun, China

**Keywords:** natural products, cardiovascular diseases, zebrafish, bioactivity, ethnopharmacology

## Abstract

Cardiovascular diseases (CVDs) are a major global health challenge, significantly impacting public health and healthcare systems. Ethnopharmacological remedies and botanical medicines are widely used for the prevention and treatment of CVDs due to their multi-component, multi-target properties. Understanding the mechanisms of these natural products is essential for developing safe and effective therapies. The zebrafish model, an emerging tool in experimental pharmacology, has been increasingly used to evaluate the pharmacological activities of natural products. This review focuses the use of zebrafish models to test the prevention and treatment effects of various CVDs, and to investigate the inhibition of hyperlipidemia, thrombosis, the progress of heart failure, and the promotion of angiogenesis and toxicity. Emphasizing its experimental advantages-including the transparency characteristics of the body, its applicability to the analysis of multiple samples, and its support for real-time monitoring-aims to reveal its potential value in combining traditional cognition with contemporary pharmacological testing capabilities. The literature summary provides strong evidence for the cognitive improvement of the zebrafish system in clarifying the efficacy of natural ingredients in the cardiovascular field.

## 1 Introduction

Cardiovascular diseases (CVDs) are a leading global cause of death, contributing significantly to morbidity and mortality worldwide (Bournele and Beis, 2016). These include various heart and blood vessel conditions such as coronary artery disease, heart failure, thrombosis, hyperlipidemia, atherosclerosis, and vascular damage (Flora and Nayak, 2019). CVDs treatment mainly involves pharmacological intervention and surgery (Li, 2024). Although these therapies can relieve symptoms, long-term use of drugs can bring the risk of drug dependence and adverse reactions.

Traditional medicine (ethnopharmacology) has a long history of use. With people’s increasing interest in traditional medicine, more and more research is exploring the potential of traditional therapies and their natural compounds in the treatment of CVDs (Zhong et al., 2024). Traditional medicine emphasizes the treatment of CVDs by improving blood circulation, enhancing overall energy, enhancing heart function, and reducing inflammation or toxins to restore body balance. In recent years, modern pharmacological research has gradually revealed the role of certain natural products in cardiovascular treatment. For example, phytochemicals such as tanshinone from *Salvia miltiorrhiza* Bunge [Lamiaceae*; Salviae miltiorrhizae* radix et rhizoma], notoginsenoside from *Panax notoginseng* (Burkill) F.H.Chen [Araliaceae*; Notoginseng* radix et rhizoma], and ginsenosides from *Panax ginseng* C.A.Mey. [Araliaceae*; Ginseng* radix et rhizoma] regulate cardiovascular function through multi-target and multi-pathway mechanisms (Fan W. X. et al., 2020; Zeng et al., 2023; Dabbaghi et al., 2024). Furthermore, the active components of these natural products have shown significant therapeutic effects in anti-inflammatory, antioxidant, lipid-lowering, and cardiovascular protection, and are widely used in the treatment of CVDs. Overall, as a vast repository of knowledge, traditional medicine holds significant value for cardiovascular disease research and warrants further investigation.

Preclinical studies of CVDs commonly use cell and mammalian models to evaluate the efficacy of therapeutic candidates (Camacho et al., 2016; Lippi et al., 2020). It is possible to replicate the pathological processes of many CVDs *in vivo* by using rats and mice as model organisms (Garbern et al., 2013). Cell models are easy to use, sensitive, and convenient for studying drug mechanisms. However, factors such as cost, trial duration, and the need for continuous, holistic *in vivo* assessment of pathological changes and drug safety and efficacy have made the zebrafish model a powerful tool for investigating how natural compounds regulate CVDs (Bournele and Beis, 2016).

**FIGURE 1:**
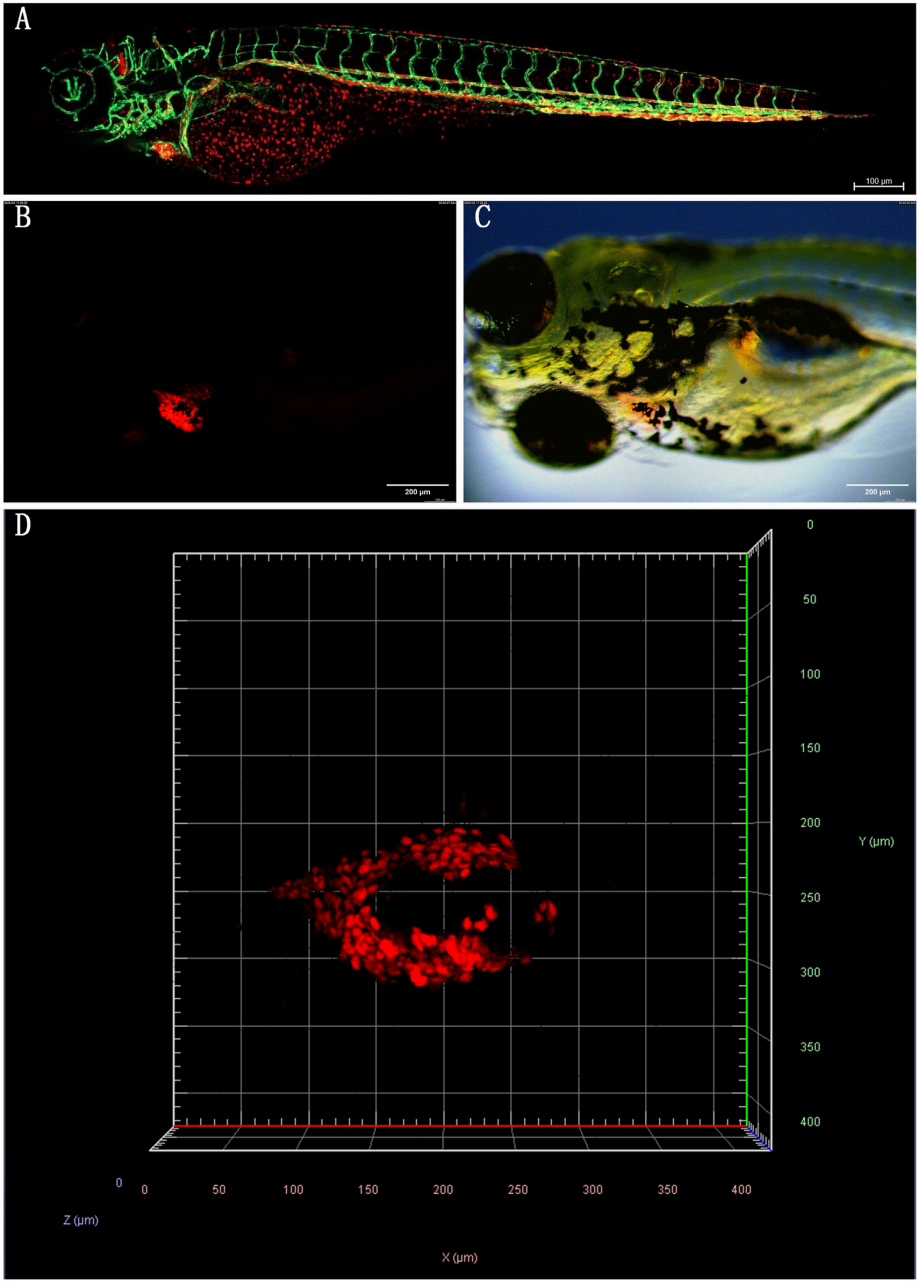
**(A)** Tg (gata1:DsRed/kdrl:eGFP) double transgenic zebrafish (2dpf): Confocal images of red fluorescence in blood cells and green fluorescence in vasculature are merged. **(B)** Tg (myl7:DsRed) transgenic zebrafish (4dpf) was observed under a fluorescence microscope. **(C)** The merge images of Tg (myl7:DsRed) transgenic zebrafish under bright and fluorescence field. **(D)** Confocal microscopy image of the heart of Tg (myl7:DsRed) transgenic zebrafish. Head is to the left. (All images in this figure are original images, taken by the author in the laboratory of Changchun University of Chinese Medicine using Leica fluorescence microscope and confocal microscope. Zebrafish were anesthetized with MS-222, fixed in low-melting agarose gel, and immobilized under the microscope. Images were taken under fluorescence and bright-field conditions, and the captured images were merged using Visio software for final processing).

Zebrafish (*Danio rerio*) are excellent models for biological and pharmacological research because they are transparent at an early stage, can dynamically observe organs and cells, have low cost, high throughput, and short experimental cycles (Gao et al., 2017). Their cardiovascular system has many structural and functional similarities with mammals. Within 24 h after fertilization (hpf), the circulatory system, including the sinuses, atria, ventricles, and bulbous arteries, is visible under a microscope. Fluorescent transgenic zebrafish have further enhanced the ability to study the cardiovascular system *in vivo*. For example, the Tg (gata1:DsRed/kdrl:eGFP) double transgenic zebrafish express eGFP (green fluorescent protein) in vascular endothelial cells, while red blood cells express DsRed (red fluorescent protein). This zebrafish was imaged using a confocal microscope to produce a composite image of the cardiovascular system (Figure 1A), in which the red fluorescently labeled red blood cells are clearly visible in the blood vessel network, providing details of blood distribution and circulation. Simultaneously, the green fluorescence of the blood vessel network reveals the complex structure of the zebrafish cardiovascular system, including the aorta, veins, and caudal vein vascular plexus. This model provides a comprehensive view of red blood cell flow and cardiovascular architecture. It has been used to study tumor vasculature dynamics (Zhong et al., 2023). Additionally, Tg (myl7:DsRed) transgenic zebrafish express DsRed in cardiomyocytes, with bright red fluorescence outlining heart morphology and position, providing an important basis for evaluating the contractile and diastolic function of the heart (Figures 1B–D).

**FIGURE 2:**
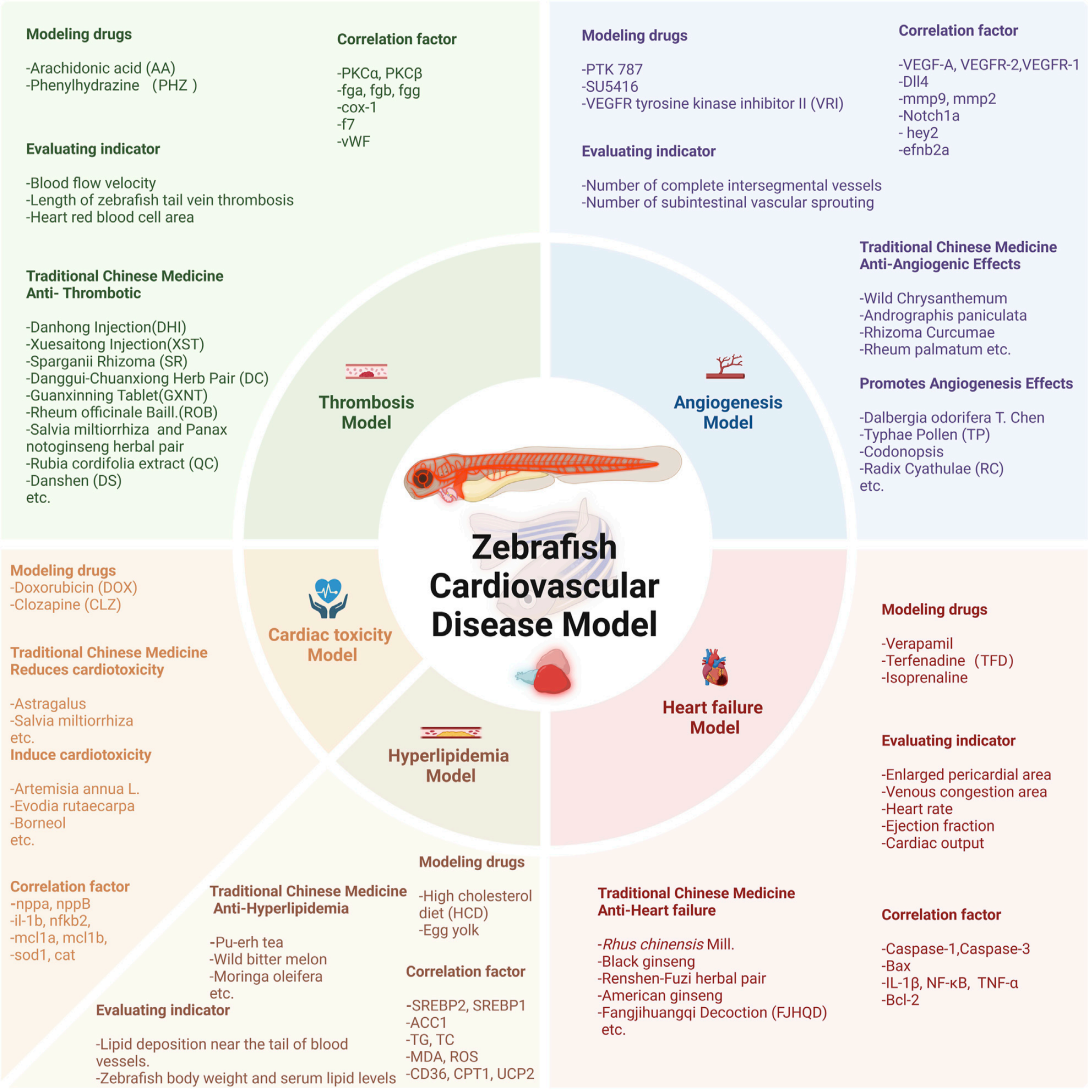
Overview of the zebrafish CVDs model and the natural products with therapeutic effects. This figure was originally created by the author using BioRender (https://biorender.com) and does not reproduce any published material.

This paper reviews the application of the zebrafish model in studying the effects and mechanisms of natural products on cardiovascular health over the past two decades, spanning from 2002 to 2025. Literature searches were conducted across multiple databases, including PubMed, Web of Science, and Google Scholar, using keywords such as “zebrafish,” “cardiovascular disease,” “natural medicine,” and “pharmacological activity.” Studies were included if they utilized zebrafish models to investigate the effects of natural compounds or extracts on CVDs—such as heart failure, hyperlipidemia, and thrombosis—provided they featured clear experimental designs and reliable data. Publications that did not employ zebrafish models, involved synthetic compounds, or presented incomplete data were excluded. We comprehensively reviewed zebrafish models assessing the effects of natural products on cardiovascular function and pathology (Figure 2). Additionally, this study aims to encourage researchers to focus on zebrafish models, promoting the development and utilization of effective models to identify candidate compounds from phytomedicines and natural products for modulating CVDs, ultimately advancing drug and health product development.

## 2 Evaluation of natural product bioactivities in various zebrafish CVDs models

### 2.1 Thrombosis model

Thrombotic disorders impact human health by affecting tissues and organs. Disrupted downstream circulation due to embolism and thrombosis can lead to ischemia and necrosis, contributing to numerous significant cardiovascular and cerebrovascular diseases (Zhang et al., 2020). The coagulation cascade promotes fibrinogenesis through the sequential activation of coagulation components. The fibrinogen α chain gene (FGA) is involved in fibrin formation, coagulation factor VII (F7) mediates the extrinsic coagulation pathway, and cyclooxygenase 1 (COX1) catalyzes the conversion of arachidonic acid into thromboxane A2, which activates platelets and promotes thrombosis. These genes and their products play critical roles in disease progression (Ishikawa et al., 2007; Hellmann et al., 2015). The zebrafish model shares similar hemostatic and thrombotic mechanisms with mammals, as it contains the same key proteins involved in platelet adhesion, activation, aggregation, and release. Moreover, zebrafish coagulation factors and platelet receptors respond to clinically used antiplatelet and anticoagulant drugs (Weyand and Shavit, 2014). Thus, zebrafish models can be effectively used to study thrombus formation in CVDs.

**FIGURE 3:**
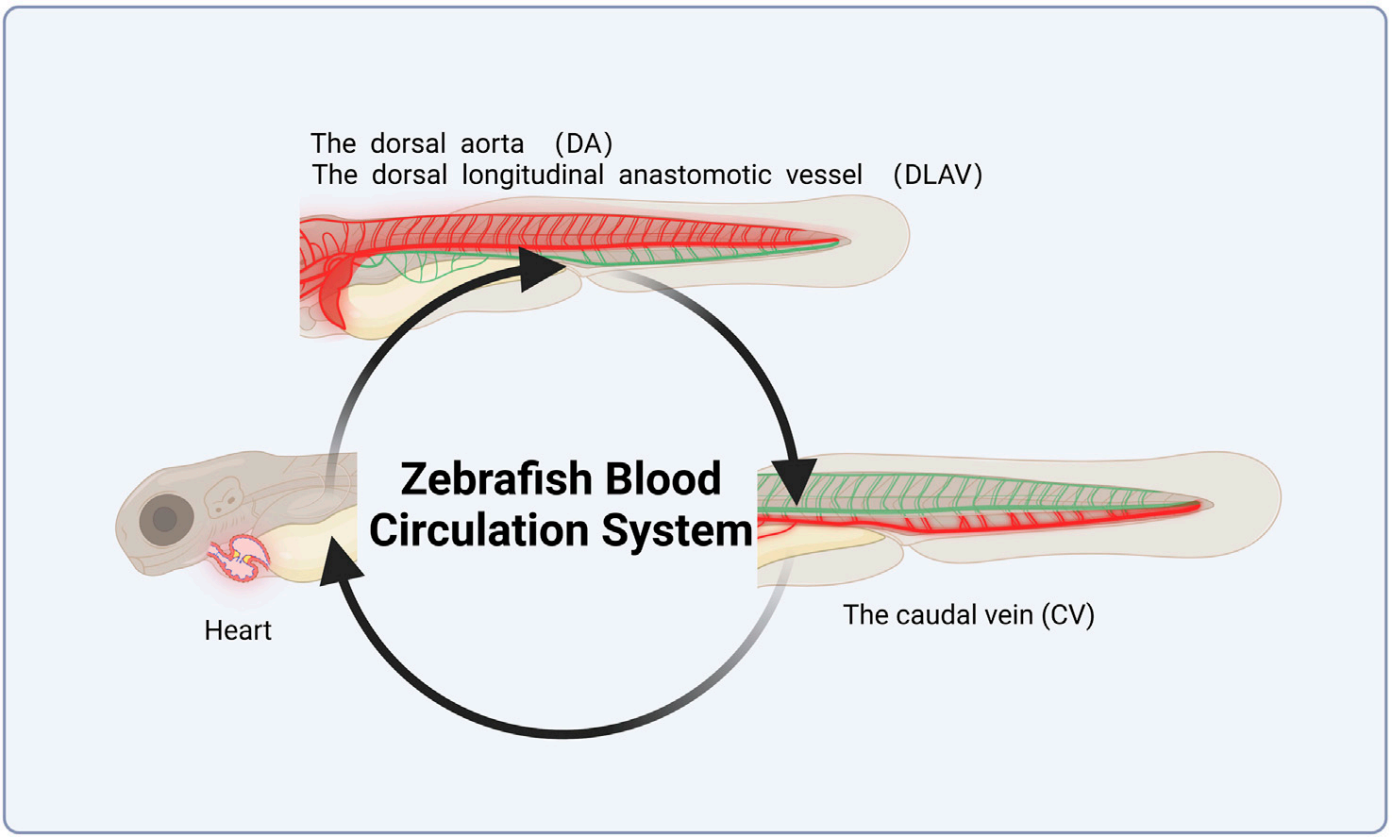
Schematic diagram of zebrafish blood circulation system. This figure was originally created by the author using BioRender (https://biorender.com) and does not reproduce any published material.

The developmental stage of zebrafish larvae features a closed circulatory system, comprising the heart, dorsal aorta (DA), dorsal longitudinal anastomotic vessel (DLAV), and caudal vein (CV), which together form a complete blood circulation circuit (Figure 3). Thrombosis in the zebrafish tail vein reduces the number of cardiac red blood cells (RBCs). Most studies utilize o-dianisidine to stain wild-type AB zebrafish. Tail vein thrombosis in zebrafish is inversely correlated with cardiac RBCs intensity, with the length and area of the thrombus used to assess its severity (Xu et al., 2021). The model is considered successful when image analysis and statistical data show a reduction in RBCs following medication intervention. Some studies have employed transgenic zebrafish strains, such as Tg (LCR-GFP), to observe blood flow and blood flow velocity under a fluorescent microscope, using these metrics to evaluate thrombosis severity (Qi et al., 2017; Li J. et al., 2020).

Arachidonic acid (AA) and phenylhydrazine (PHZ) are commonly used to model thrombosis in zebrafish. AA activates platelets through the generation of TXA2, which binds to fibrinogen via GPIIb/IIIa receptors, promoting platelet aggregation and thrombosis in zebrafish (Warner et al., 2011). Table 1 summarizes the natural products with antithrombotic activity studied in the AA-induced zebrafish model. For instance, salvianolic acid B, danshensu, lithospermic acid, rosmarinic acid, P-coumaric acid, and hydroxysafflor yellow A—key active metabolites in danhong Injection, a traditional Chinese medicine—alleviate blood flow obstruction in the zebrafish tail vein and restore heart RBCs count (Qi et al., 2017). Furthermore, the primary bioactive metabolites of *Xuesaitong Injection*, including notoginsenoside R1, ginsenosides Rg1, Rb1, and Rd, downregulate coagulation-related genes such as fga and ptgs2b, reducing inflammation. This approach prevents thrombus formation by inhibiting platelet aggregation, restoring cardiac RBCs density, and reducing abnormal erythrocyte accumulation in the caudal vein (Ma et al., 2021). Additionally, *Danggui-Chuanxiong Herb Pair* (DC), comprising *Angelica sinensis* (Oliv.) Diels [Apiaceae; *Angelicae* sinensis radix] and *Ligusticum chuanxiong* Hort. [Apiaceae; *Ligustici Chuanxiong* rhizoma], enhances myocardial erythrocyte density and suppresses AA-induced thrombosis (Pang et al., 2024). A network pharmacology study identified 24 DC-derived antithrombotic compounds and 89 targets, mainly involved in inflammatory pathways, angiogenesis and hormone regulation (Zhang et al., 2021). Moreover, ferulic acid, protocatechuic acid, and P-coumaric acid derived from the ethanol extract of *Sparganium stoloniferum* Buch.-Ham. ex Juzepczuk [*Sparganiaceae;* Sparganii Rhizoma] have been shown to promote cardiac erythrocyte recovery in a dose-dependent manner (Xu et al., 2021).

**TABLE 1:** Evaluation of natural products for antithrombotic effects using zebrafish models.

Source	Extracts/Compounds	Model	Finding	Reference
Danhong Injection(DHI) (*Salvia miltiorrhiza* Bunge [Lamiaceae; *Salviae miltiorrhizae* radix et rhizoma] and *Carthamus tinctorius* L. [Asteraceae; *Carthami tinctorii* flos])	Danshen (DS) and Honghua (HH) Salvianolic acid B (SaB), Danshensu (DSS), Lithospermic acid (LA) Rosmarinic acid (RA) form DS P-coumaric acid (pCA) Hydroxysafflor yellow A (HSYA) from HH	Wild-type Tuebingen (TU) strain and Tg (LCR-GFP) 3dpf zebrafish larvae Arachidonic acid (AA) (40 μM, 1 h) Aspirin (22.5 g/mL) as positive control Stain with o-dianisidine	DHI components (DS, HH, SaB, DSS, LA, RA, pCA, HSYA) inhibit tail vein obstruction; restore cardiac red blood cells (RBCs)	Qi et al. (2017)
Xuesaitong Injection(XST) (*Panax notoginseng* (Burkill) F.H.Chen [Araliaceae; *Notoginseng* Radix et Rhizoma])	Notoginsenoside R1(R1), ginsenoside Rg1(Rg1), ginsenoside Rb1(Rb1) and ginsenoside Rd (Rd)	Wild-type Tuebingen (TU) strain and Tg (LCR-GFP) 3dpf zebrafish larvae Arachidonic acid (AA) (40 μM, 1 h) Stain with o-dianisidine	XST components (R1, Rg1, Rb1, Rd) restore cardiac RBCs; reduce tail vein RBCs accumulation; inhibit platelet aggregation (FGA↓); anti-inflammatory	Ma et al. (2021)
*Sparganium stoloniferum* Buch.-Ham. ex Juzepczuk [Sparganiaceae; *Sparganii* Rhizoma] (SR)	Ferulic acid, protocatechuic acid and P-coumaric acid	Wild-type AB strain 3dpf zebrafish larvae Arachidonic acid (AA) (100 μmol/L,1 h) Stain with o-dianisidine	SR ethanol extract and active compounds (ferulic acid, protocatechuic acid, P-coumaric acid) dose-dependently restore cardiac RBCs; antithrombotic	Xu et al. (2021)
Danggui-Chuanxiong Herb Pair (DC) (*Angelica sinensis* (Oliv.) Diels [Apiaceae; Angelicae sinensis radix] and *Ligusticum chuanxiong* Hort. [Apiaceae; *Ligustici Chuanxiong* rhizoma])	Ferulic acid (DC2), ligustilide (DC7), and levistilide A (DC17)	Wild-type AB strain 3 dpf zebrafish larvae After 6 h of drug pretreatment, Arachidonic acid (AA) (80 μM) for 1 h Stain with o-dianisidine	DC components (DC2, DC7, DC17) increase cardiac RBCs density; antithrombotic, anti-inflammatory, antioxidant, angiogenic	Zhang et al. (2021)
Guanxinning Tablet (GXNT) (*Salvia miltiorrhiza* Bunge [Lamiaceae; *Salviae miltiorrhizae* radix et rhizoma] and *Ligusticum striatum* DC. [Apiaceae; *Ligustici striati* rhizoma])	Cryptotanshinone and Senkyunolide I	Tg (LCR: EGFP) and Tg (CD41: EGFP) 84hpf zebrafish larvae Phenylhydrazine (PHZ) (0.75 µM, 12 h)	GXNT, Cryptotanshinone and Senkyunolide I promote circulation; inhibit thrombosis via MDA↓, TNF-α↓, cox1↓, f7↓, fgb↓ The combination of Cryptotanshinone and Senkyunolide I has better antithrombotic effect	Li et al. (2021)
*Rheum officinale* Baill. (ROB) (*Rheum officinale* Baill. [Polygonaceae; *Rhei* radix et rhizoma])	Emodin, aloe-emodin and physcion	Wild-type AB strain 72 hpf zebrafish larvae Phenylhydrazine (PHZ) (10 μM, 24 h) Stain with Reactive oxygen species (ROS) and Dichlorodihydrofluorescein diacetate (DCFH-DA)	ROB (emodin, aloe-emodin, physcion) decrease the staining intensity/area of ROS; improve platelet adhesion/aggregation; regulate NOS2/NOS3, arginine/glutamate pathways	Zhang et al. (2022b)
(Danshen, DS) and (Sanqi, SQ) herbal pair (DS–SQ) (*Salvia miltiorrhiza* Bunge [Lamiaceae; *Salviae miltiorrhizae* radix et rhizoma] and *Panax notoginseng* (Burkill) F.H.Chen [Araliaceae; *Notoginseng* radix et rhizoma])	Danshensu (DSS), protocatechuic acid (PRAC), rosmarinic acid (MA), lithospermic acid (LA), salvianolic acid B (SAB) and salvianolic acid A (SAA) of DS ginsenoside Rg1 (GRg1) and ginsenoside Rb1 (GRb1) of SQ	Wild-type AB strain 4dpf zebrafish larvae Phenylhydrazine(PHZ) 1.5 μM, 12 h Stain with o-dianisidine	DS-SQ (10:1 ratio) ↑ cardiac RBCs, ↓ tail vein aggregation Among the components of DS-SQ, MA, LA and SAB had the most significant antithrombotic activity PKCα↓, PKCβ↓, fga↓, fgb↓, fgg↓, vWF↓	Yin et al. (2020)
*Rubia cordifolia* L. [*Rubiaceae; Rubiae* radix]	*Rubia cordifolia* extract (QC) Anthraquinones, arborinane-type triterpenoids, and cyclic hexapeptides	Wild-type AB strain 2 dpf zebrafish embryos Phenylhydrazine(PHZ) (1.5 µmol/L, 24 h) Stain with o-dianisidine	EC_50_ (QC)= 52.66 µg/mL QC treatment group can enhance the staining intensity of cardiac RBCs and reduce tail vein thrombosis. kdrl↑, flt-1↑, vWF↑, VEGF-A↑	Chen et al. (2018b)
Danshen (DS) (The dry root of *Salvia miltiorrhiza* Bunge [Lamiaceae; *Salviae miltiorrhizae* radix et rhizoma])	Salvianolic acid B (SAB), lithospermic acid (LA), rosmarinic acid (MA), and luteolin-7-O-β-D-glucoside (LG)	Wild-type AB strain 78 hpf zebrafish larvae 15 µM (±)-epinephrine hydrochloride(AH) or 1.5 µM Phenylhydrazine (PHZ) for 16 h or 24 h Stain with o-dianisidine	DS components (SAB, MA, LA, LG) ↓ thrombus formation; restore RBCs intensity; bound thrombosis-related proteins well (molecular docking)	Tang et al. (2021)
Herb pair Danshen-Chuanxiong(*Salvia miltiorrhiza* Bunge [Lamiaceae; *Salviae miltiorrhizae* radix et rhizoma] and *Ligusticum chuanxiong* Hort. [Apiaceae; *Ligustici Chuanxiong* rhizoma])	Danshen-Chuanxiong (2:1) water and ethanol extract (key compounds include salvianolic acid B, ligustilide, rosmarinic acid, chlorogenic acid, ferulic acid)	Wild-type AB strain 2dpf zebrafish embryos ponatinib (1 μg/mL) for 24 h Aspirin (30 μg/mL) as positive control	Danshen-Chuanxiong (2:1) inhibits PHZ-induced thrombosis (dose-dependent), superior to single herbs TNF-α↓,IL-1β↓	Pang et al. (2024)
Dang-Gui-Si-Ni (DGSN) decoction(*Angelica sinensis* (Oliv.) Diels [Apiaceae; *Angelicae sinensis* radix], *Cinnamomum cassia* (L.) J. Presl [Lauraceae; *Cinnamomi* ramulus], *Paeonia lactiflora* Pall. [Paeoniaceae; *Paeoniae* radix alba], *Asarum heterotropoides* F. Schmidt [Aristolochiaceae; *Asari* radix et rhizoma], *Akebia trifoliata* (Thunb.) Koidz. [Lardizabalaceae; Akebiae caulis], *Glycyrrhiza uralensis* Fisch. [Fabaceae; *Glycyrrhizae* radix et rhizoma] and *Ziziphus jujuba* Mill. [Rhamnaceae; Jujubae fructus])	*Angelicae sinensis Radix* (ASR)*, Cinnamomi Ramulus* (CR)*, Paeoniae* *Radix Alba* (PRA)*, Asari Radix et Rhizoma* (ARR)*, Akebiae Caulis* (AC) *Glycyrrhizae Radix et Rhizoma* (GRR) *and Jujubae Fructus* (JF)	Wild-type AB strain 5dpf zebrafish larvae Punatinib 5 mg/mL for 18 h Aspirin(60 μg/mL) as positive control	DGSN decoction (500 μg/mL) can significantly increase the cardiac outputˎ enhance the blood flow and cardiac output in thrombotic model zebrafish FII↓, FVII I↓, FIX I↓, FX I↓	Li et al. (2024d)
*Toddalia asiatica (L.)* Lam. [Rutaceae; *Toddaliae* radix] (TA)	The extracts of TA	Wild-type AB strain 3dpf zebrafish larvae PHZ (1.5 μM) for 24 h aspirin (25 μg/mL) was used as positive control	Increase the staining intensity of RBCs in the zebrafish heart and reduce thrombosis in the tail vein in a dose-dependent manner TA inhibited coagulation factor III (TF) expression via the PI3K-Akt-NF-κB signaling pathway, resulting in antithrombotic effects	Yang et al. (2024)
*Raw Moutan Cortex* (RMC) (*Paeonia × suffruticosa* Andrews [Paeoniaceae; *Paeoniae* radix et cortex])	The extracts of Moutan cortex	Wild-type AB strain 3 dpf zebrafish larvae 80 μM AA for 1.5 h	RMC significantly reduced the area of caudal vein thrombosis in zebrafish Inhibiting the factors related to coagulation-MARK inflammation pathway il-6↓, COX-2↓, iNOS↓, TNF-α↓, ERK↓, JNK↓, p38↓	Huang et al. (2024)
*Trichosanthes kirilowii* Maxim. [Cucurbitaceae; *Trichosanthis* pericarpium] (TP; Gualoupi)	“HaiShi GuaLou” and “WanLou”	Wild-type AB strain 3 dpf zebrafish larvae 1.5 μmol/L PHZ (Aladdin, China) for 24 h Aspirin (5.625 μg/mL) as positive control	TP (50–200 μg/mL) dose-dependently reduced tail vein thrombus and increased heart RBCs staining intensity PKCα↓, PKCβ↓, fga↓, Fgg↓, F2↓, F7↓, PTGS1↓, vWF↓,PKCβ↓	Xia et al. (2024)
*Crocus sativus* L. [Iridaceae; *Croci sativae* stigma]	Crocin-4	Wild-type AB strain 3dpf zebrafish larvae 80 μmol/L arachidonic acid(AA) for 24 h Aspirin group(22.5 μg/mL) as positive control	Reduced RBCs accumulation in zebrafish tail, effectively improved thrombosis in zebrafish larvae induced with AA. PAI-1↓, TF↓, f2↓, f7↓, fgα↓, tbxas1↓	Xu et al. (2024)
*Salvia miltiorrhiza* Bunge [Lamiaceae; *Salviae miltiorrhizae* radix et rhizoma] (Danshen, DS) *and Paeonia veitchii* Lynch [Ranunculaceae; *Paeoniae* radix rubra]. (Chishao, CS) herbal pair (DS-CS)	The extract of DS-CS Salvianolic Acid A (SAA) and Paeoniflorin (PF)	Wild-type AB strain 24 hpf zebrafish embryos 1.5 μM PHZ for 48 h Aspirin 25 μg/mL was used as positive control	DS-CS extract can inhibit PHZ-induced zebrafish thrombosis in a dose-dependent manner EGFR↑, F10↓, SRC↓	Rao et al. (2024)

This summary provides information on the regulatory effects of drugs or natural products on thrombosis, using wild-type and transgenic zebrafish as experimental models. “↑” or “↓” illustrates the regulatory effects of drugs or natural products on different indicators. “↑” indicates increase or upregulation; “↓” indicates reduce or downregulation. dpf: days post-fertilization; hpf: hours post-fertilization. EC_50_: median effect concentration.

On the other hand, PHZ increases thrombin production, exonerates phosphatidylserine in the RBCs membrane, and produces superoxide radicals, which oxidize thrombin. PHZ also induces inflammation, causes endothelial dysfunction, and promotes a state of hypercoagulation, which ultimately leads to thrombosis (Zhu et al., 2016). Pharmacological therapies targeting these mechanisms have been explored. A PHZ-induced zebrafish thrombosis model demonstrated the synergistic antithrombotic effects of cryptotanshinone and senkyunolide I (Li J. et al., 2020). Cryptotanshinone inhibits cox1 and TNF-α, while ligustrinone I targets f7 and fibrinogen β chain (fgb) expression in the extrinsic coagulation pathway. These compounds collectively enhance RBCs and platelet circulation, reduce peripheral blood flow restriction, and modulate endogenous platelet activation and coagulation cascades. Furthermore, the TCM formula *Dang-Gui-Si-Ni Decoction* can regulate a variety of coagulation factors and has anticoagulant effects. It increases the cardiac output and blood flow rate of zebrafish, while significantly reducing the expression of coagulation factor II (FII), VII (FVII), IX (FIX) and X (FX) RNA *in vivo* (Li Y. et al., 2024). When evaluating the yolk sac thrombosis area in zebrafish larvae, the *P. notoginseng-Danshen herbal pair* (DS-SQ) exhibited the most potent antithrombotic activity (Yin et al., 2020). Several studies have found that compounds such as luteolin-7-O-β-D-glucoside, rosmarinic acid, salvianolic acid B, and lithospermic acid bind strongly to zebrafish thrombosis proteins. These active compounds significantly reduce PHZ-induced tail vein thrombosis and enhance cardiac RBCs levels (Tang et al., 2021). The aqueous extract of *Rheum officinale* Baill (ROB) mitigates thrombus formation, oxidative stress, and platelet adhesion by upregulating nitric oxide synthase 3 (NOS3) expression and modulating arginine biosynthesis pathways (Zhang Y. R. et al., 2022). Lastly, the extract of *Toddalia asiatica* (L.) Lam. [Rutaceae; *Toddaliae* radix] demonstrates notable antithrombotic effects in zebrafish through regulation of the PI3K-Akt-NF-κB signaling pathway (Yang et al., 2024).

While these models excel at high-throughput screening and allow real-time visualization of thrombus formation, the absence of platelets structurally identical to those in mammals—combined with differences in coagulation factor composition—requires cautious interpretation when extrapolating to human thrombotic diseases. We recommend that promising candidates identified in zebrafish should undergo validation in rodent models before clinical consideration.

### 2.2 Hyperlipidemia model

Hyperlipidemia, a chronic disturbance in lipid metabolism, elevates the risk of atherosclerosis, coronary heart disease, and other CVDs (Bozkurt et al., 2016). It involves the complex regulation of several pathways and key molecules. For example, blood lipid balance is controlled by the activation of the SREBP and PPAR pathways. Acetyl-CoA carboxylase 1 (ACC1) plays a vital role in fatty acid synthesis, which affects plasma lipid levels. Triglycerides (TG) and cholesterol (TC) are the main components of blood lipids and are direct indicators of lipid metabolism. These pathways regulate intracellular lipid synthesis, fatty acid metabolism, cholesterol transport, and inflammation. In addition, lipid metabolism is regulated by factors such as cd36, CPT1, and UCP2, which regulate energy metabolism, fatty acid transport, and β-oxidation.

Zebrafish have similarities with humans in lipid metabolism and have significant homology in apolipoproteins (Otis et al., 2015). High-density lipoprotein (HDL), low-density lipoprotein (LDL), and very low-density lipoprotein (VLDL) have been identified in zebrafish plasma (Stoletov et al., 2009). Therefore, zebrafish is a valuable model for studying the pathophysiology of hyperlipidemia and testing potential therapies. In hyperlipidemia studies using natural products, zebrafish models are usually induced by a high-fat diet (Table 2). For example, feeding 0.1% egg yolk of zebrafish for 48 h induces hyperlipidemia, and treatment with Pu’er tea extract significantly reduces blood lipid levels (Zhou et al., 2015). Another study using a 4% cholesterol diet and red fluorescent lipids (10 μg/g) in 5-day-old Tg (fli1:EGFP) zebrafish demonstrated that Dendrobium huoshanense polysaccharide (DHP) reduced vascular lipid deposition, improved lipid metabolism, and alleviated oxidative stress and inflammation by enhancing SOD activity, inhibiting neutrophil recruitment, and decreasing TC/TG/MDA/ROS levels (Fan X. C. et al., 2020). Additionally, a hyperlipidemia model using ground Artemia nauplii and egg yolk powder confirmed Bergenin’s effectiveness in reducing hepatic and vascular lipid accumulation in high-fat-fed larvae (Zhang et al., 2023).

**TABLE 2:** Evaluation of natural products for hypolipidemic effects using zebrafish models.

Source	Extracts/Compounds	Model	Finding	Reference
Pu-erh tea(*Camellia sinensis* (L.) Kuntze var. *assamica* (Masters) Kitamura [Theaceae; *Camelliae sinensis* folium post-fermentatum])	Pu-erh tea extracts	Wild-type AB strain 5 dpf zebrafish larvae Feed with 0.1% egg yolk for 48 h Oil Red O (ORO) in zebrafish gut, vessel and vena caudalis	Pu’er tea extract can significantly reduce lipid accumulation in the tail vein of hyperlipidemic zebrafish	Zhou et al. (2015)
*Eupolyphaga sinensis* Walker [Blattidae; *Eupolyphagae* sinensis] (ESW)	Active peptides from E. *sinensis* Walker (APE)	Wild-type AB strain 5 dpf until 9 dpf zebrafish larvae Feed with Gemma Micro 75 ZF mixed with 20% freeze-dried egg yolk powder from 7dpf to 9dpf On 10 dpf, Oil Red O staining in tail veins	APE reduces lipid deposition induced by HFD consumption in zebrafish	Dong et al. (2024a)
*Salvia miltiorrhiza* Bunge [Lamiaceae; Salviae miltiorrhizae radix et rhizoma] and *Carthamus tinctorius* L. [Asteraceae; *Carthami tinctorii* flos]	Protocatechualdehyde (PCA) and Hydroxysafflor yellow A (HSYA)	Wild-type AB strain 5 dpf zebrafish larvae Feed high-cholesterol diet (HCD) (4% weight per weight cholesterol added to egg yolk powder) for 48 h Oil Red O staining in the caudal vein	PCA and HSYA ↓ lipid deposition in zebrafish tail vein (combination > single) PPAR-γ↓, SREBP2↓, SREBP1, HMGCR↓, PCSK9↓, mTOR↓, C/EBPα↓, LDLR↑, AMPK↑, HNF-1α↓, FoxO3a↑	Lin et al. (2024)
Wild bitter melon (WBM;*Momordica charantia* L. var. abbreviata Ser. [Cucurbitaceae; *Momordicae charantiae* folium])	*Momordica charantia* leaf extract(3β,7β,25-trihydroxycucurbita-5,23-dien-19-aL (TCD) fraction of WBM leaf extract)	Wild-type AB strain Four to six-month-old adult zebrafish Overfed with 13 mg of artemia (60% protein, 7% fat, 5% ash, and 8% water) and 13 mg of normal fish flakes per day for 8 weeks	Reduced plasma TG and total cholesterol Decreased hepatic lipid deposition and restored hepatocyte morphology SREBP↓, PPAR↓, ACC1↓, FASN↓, CD36↓, CPT1↓, UCP2↓	Wu et al. (2021)
*Dendrobium huoshanense* C. Z. Tang et S. J. Cheng [Orchidaceae; *Dendrobii huoshanensis* caulis] (DH)	*Dendrobium huoshanense* C. Z. Tang et S. J.Cheng polysaccharide (DHP)	Tg (fli1: EGFP) 5 dpf zebrafish larvae Feed with 4% cholesterol (supplemented with 10 μg/g of red fluorescent lipid) diet for 10 days	DHP ↓ lipid deposits near vessels; ↑ lipid metabolism; improve endothelial dysfunction	Fan X. C. et al. (2020)
*Moringa oleifera* Lam. [Moringaceae; *Moringae oleiferae* folium et semen]	Alcoholic extract of *Moringa oleifera* seeds	Wild-type AB strain 5 dpf zebrafish larvae Feed with 0.3% egg yolk aqueous solution for 2 days Oil red O staining in the tail vein of zebrafish	*Moringa oleifera* seeds extract ↓ vascular ORO staining TG↓, TC↓	Yuan et al. (2022)
*Mytilus edulis* Linnaeus [Mytilidae; *Mytili edulis* concha]	Mussel-derived plasmalogens (PLs)	Wild-type AB strain 1-year-old adult zebrafish Feed with mixing cholesterol powder (human food grade) with normal fish feed in 4% ratio for 6 weeks	Pls was effective in normalizing zebrafish body weight and serum lipid levels Reduce hepatic TC/TG levels	Feng et al. (2023)
*Saxifraga melanocentra* Franch. [Saxifragaceae; *Saxifragae melanocentrae* herba]	Bergenin	Wild-type AB strain 20dpf zebrafish larvae Feed ground A. nauplii (2 mg/fish/day) and egg yolk powder (1.5 mg/mL) continuous feeding for 7 days, 6 h a day Oil Red O staining in the tail vessels and liver	Reduce hepatic/vessel lipid deposition Reduce TG, TC and low-density lipoprotein cholesterol (LDL-c) levels; increase high-density lipoprotein cholesterol (HDL-c) levels FASN↓, SREBF1↓, HMGCRα↓, RORα↓, LDLRα↓, IL-1β↓, TNF↓, IL-4↑	Zhang et al. (2023)
Danggui Shaoyao San (DSS) (*Angelica sinensis* (Oliv.) Diels [Apiaceae; *Angelicae sinensis* radix] and *Paeonia lactiflora* Pall. [Paeoniaceae; *Paeoniae alba* radix]	Compounds of DSS	Wild-type AB strain 4dpf zebrafish larvae, adult zebrafish Feed a diet containing 0.1% egg yolk powder Oil Red O staining in both larval fish tail vessels	DSS impedes weight gain; reduces liver lipid accumulation; enhance locomotor activity; regulate lipid metabolism PPARγ↑, ABCA1↑	Wang et al. (2023)
*Fallopia multiflora* (Thunb.) Harald. [Polygonaceae; *Polygoni multiflori* radix] (Heshouwu) and *Rheum palmatum* L. [Polygonaceae; *Rhei* radix et rhizoma] (Dahuang)	Emodin (EM)	Wild-type AB strain and Tg (flil: eGFP) 5 dpf zebrafish larvae Treated with 4% high-cholesterol diet (mixing a diethyl ether solution of cholesterol with normal feed to get 4% (w/w) cholesterol in the diet after diethyl ether evaporation, and additionally added with 10 μg/g of cholesteryl BODIPY® 542/563-C11 in the dark.) for 10 days Oil red O staining and H&E staining	EM reduced vascular/hepatic lipid deposition, alleviated hepatic histological damage, and inhibited vascular neutrophil inflammation EM can ameliorate abnormal lipid levels involved in TC, TG, LDL-C and HDL-C levels AMPKα↑, LDLR↑, ABCA1↑ ABCG1↑, SREBP-2↓, PCSK9↓, HMGCR↓	He et al. (2021)
Mountain-cultivated *Panax ginseng* C. A. Mey. [Araliaceae; *Ginseng* radix et rhizoma].(MCG)	A novel glycopeptide (APMCG-1) of MCG residue	Tg (kdrl:EGFP) 5 dpf zebrafish larvae 10% high-fat diet for 8 h a day, 3% glucose solution high sugar solution for 16 h a day, for 4 consecutive days	APMCG-1 can significantly reduce blood lipid levels (such as triglycerides, total cholesterol, etc.) in zebrafish	Li et al. (2025)
*Rosa sterilis* S. D. Shi [Rosaceae; *Rosae sterilis* fructus]	R.*sterilis* total flavonoid extract (RS)	Melanin allele-mutant albino zebrafish larvae Feed egg yolk powder Oil red O staining in tail 0.081 µg/mL lovastatin as positive control	RS ↓ lipid integral optical density (IOD) (dose-dependent, at 30 μg/mL) The lipid reduction rate RS (41%) > lovastatin (26%) Key targets: HSP90AA1, PPARA, MMP9; regulation of adipocyte lipolysis and lipid metabolism pathways	Wu et al. (2024b)
*Vaccinium dunalianum* Wight [Ericaceae; *Vaccinii dunaliani* folium]	6′-O-Caffeoylarbutin (CA)	Albino zebrafish larvae 0.1% egg yolk to the fish water for 48 h Lovastatin (0.081 µg/mL) as positive control	CA can significantly reduce lipid deposition (IOD value) and has a good inhibitory effect on hyperlipidemia MMP9↓, RELA↓, MMP2↓, PRKCA↓, HSP90AA1↓, APP↓	Wu et al. (2024a)
*Cassia tora* L. [Fabaceae; *Cassiae torae* semen]	Aurantio-obtusin (AO)	Wild-type AB strain 5dpf zebrafish larvae 2 g/L egg yolk to the fish water for 72 h	AO reduced TC, TG levels and improved hepatic lipid vacuoles Downregulated TNF-α, IL-1β mRNA; Regulated IL-17, TNF, and AGE-RAGE signaling pathways	Zhou et al. (2024a)
Scutellaria baicalensis Georgi [Lamiaceae; Scutellariae baicalensis radix]	Baicalein	Wild-type AB strain 5 dpf zebrafish larvae High-fat diet (hard-boiled egg yolk) Curcumin (2.5 μM) as positive control Nile Red staining	Significantly reduced lipid accumulation Decreased TG levels PPARγ↓, aP2-a↓, aP2-b↓, SREBP-1↓, SREBP-2↓	Seo et al. (2014)
*Curcuma longa* L. [Zingiberaceae; *Curcumae longae rhizoma*] (turmeric) and *Laurus nobilis* L. [Lauraceae; *Lauri nobilis folium*] (laurel)	Aqueous extract of turmeric or laurel	Wild-type AB strain Adult zebrafish 4% cholesterol diet	Significantly reduced body weight-to-height ratio, plasma total cholesterol, triglycerides, and blood glucose; suppressed liver injury markers (GOT and GPT) (laurel reduced GOT but increased GPT) and inflammation (IL-6); inhibited cholesteryl ester transfer protein (CETP) activity	Jin et al. (2011)

This summary provides information on the regulatory effects of drugs or natural products on hyperlipidemia, using wild-type and transgenic zebrafish as experimental models. “↑” or “↓” illustrates the regulatory effects of drugs or natural products on different indicators. “↑” indicates increase or upregulation; “↓” indicates reduce or downregulation. dpf: days post-fertilization; hpf: hours post-fertilization.

Due to the successful establishment and reliable drug response of zebrafish hyperlipidemia models, researchers have been able to investigate the lipid-lowering mechanisms of natural substances. Protocatechualdehyde (PCA) and Hydroxysafflor yellow A (HSYA), either alone or in combination, reduce lipid deposition in zebrafish tail veins and alleviate hyperlipidemia-induced liver damage (Lin et al., 2024). Active peptides from *E. sinensis* Walker (APE) suppress high-fat diet (HFD)-induced lipid accumulation in zebrafish (Dong P. P. et al., 2024). The wild bitter melon (WBM) (*Momordica charantia* L. var. *abbreviata* Ser. [Cucurbitaceae; *Momordicae charantiae* folium]) leaf extract metabolite 3β,7β,25-trihydroxycucurbita-5,23-dien-19-al (TCD) inhibits adipocyte differentiation and hypertrophy, improves hepatocyte morphology, downregulates lipid metabolism genes (CD36, CPT1, UCP2), reduces ACC1 levels, and modulates the SREBP/PPAR pathway, thereby alleviating hepatic lipid accumulation and hyperlipidemia (Wu et al., 2021). Additionally, Moringa oleifera Lam. [Moringaceae; *Moringae oleiferae folium*] alcoholic extract reduces triglyceride (TG) and cholesterol (TC) levels and decreases Oil Red O staining in hyperlipidemic zebrafish tail vasculature (Yuan et al., 2022). Emodin mitigates fat accumulation in blood vessels and liver, improves liver histology, and inhibits vascular neutrophil inflammation (He et al., 2021). Eleutheroside B, the main active compound of *Eleutherococcus senticosus* (Rupr. et Maxim.) Maxim.) [Araliaceae; *Acanthopanacis senticosi* radix et rhizoma], reduces TG, TC, and LDL-C levels, increases HDL-C, and improves glucose and lipid metabolic disorders (Dong X. et al., 2024). Aqueous extracts of *Curcuma longa* L. [Zingiberaceae; *Curcumae longae rhizoma*] and *Laurus nobilis* L. [Lauraceae; *Lauri nobilis* folium] reduce plasma cholesterol and triglycerides by inhibiting CETP activity and improve obesity, hyperglycemia, and inflammation (Jin et al., 2011). Baicalein decreases lipid accumulation and triglyceride levels in zebrafish by downregulating adipogenic genes such as PPARγ, adipose protein 2 (aP2), and SREBP (Seo et al., 2014). *Danggui Shaoyao San* reduces hepatic lipid deposition in hyperlipidemic zebrafish, enhances motor activity, and regulates lipid metabolism (Wang et al., 2023). Collectively, these studies demonstrate the lipid-lowering effects of natural products and the widespread application of the zebrafish model in hyperlipidemia drug screening and evaluation. Further details are provided in Table 2. The zebrafish model holds significant value in screening lipid-lowering metabolites. However, differences in lipoprotein metabolism between zebrafish and mammals may affect the translational relevance of lipid metabolism findings. To improve translational value, future studies should complement simple lipid staining with quantitative lipoprotein analysis, plasma and tissue lipidomics, and tracking of triglyceride and cholesterol metabolism. Combining these data with mammalian validation will help better elucidate therapeutic mechanisms and enhance the predictive relevance of zebrafish hyperlipidemia screening.

### 2.3 Heart failure model

Heart failure (HF) is characterized by cardiac hypertrophy, venous congestion, bradycardia, and reduced cardiac output. Zebrafish HF models are simple, cost-effective, and allow easy monitoring of heart function in embryos (Narumanchi et al., 2021). Zebrafish embryonic heart development begins at 5 hpf, and by 48 hpf, the atrial and ventricular structures are fully formed with a stable heart rate, enabling transparent cardiac visualization under a microscope. HF models can be induced through genetic manipulation or drug treatments (e.g., isoproterenol, aristolochic acid, verapamil) (Dong et al., 2022). After exposure to these modeling agents at 48 hpf, the embryo exhibits morphological changes, such as cardiac hypertrophy and venous congestion, as well as functional changes in heart rate and hemodynamics, which can be observed through a microscope (Singleman and Holtzman, 2012; Zhu et al., 2018). In successfully modeled HF zebrafish, the therapeutic efficacy was evaluated by monitoring morphological improvements and functional changes. The recent investigation of natural products using the zebrafish HF model is summarized in Table 3.

**TABLE 3:** Evaluation of natural products for anti-heart failure effects using zebrafish models.

Source	Extracts/Compounds	Model	Finding	Reference
*Rhus chinensis* Mill. [Anacardiaceae; *Gallae chinensis*]	Triterpenoids	Wild-type AB strain and Tg (cmlc2:EGFP) 2 dpf AB stain zebrafish 3 dpf Tg (cmlc2:EGFP) 4 mg/mL ISO 5 h Digoxin (0.8 μg/mL/1.2 μg/mL) as positive control	Ten novel and nine known triterpenoids were isolated from *Rhus chinensis* Mill. Fourteen compounds alleviated pericardial edema, five reduced impaired cardiac output (CO), and eight inhibited cardiomyocyte apoptosis	Ye et al. (2023)
Qiangxinyin formula (QXY) (*Aconitum carmichaelii* Debeaux [Ranunculaceae; *Aconiti lateralis* radix praeparata], *Atractylodes macrocephala* Koidz. [Asteraceae; *Atractylodis macrocephalae* rhizoma], *Epimedium brevicornum* Maxim. [Berberidaceae; *Epimedii* folium], *Polyporus umbellatus* (Pers.) Fr. [Polyporaceae; *Polyporus*], *Psoralea corylifolia* L. [Fabaceae; *Psoraleae* fructus], *Paeonia lactiflora* Pall. [Paeoniaceae; *Paeoniae alba* radix], *Ligusticum chuanxiong* Hort. [Apiaceae; *Chuanxiong* rhizoma], *Poria cocos* (Schw.) Wolf [Polyporaceae; *Poria*] and *Cervus nippon* Temminck or *Cervus elaphus* L. [Cervidae; *Cervi* cornu])	Benzoylmesaconine (BMA), atractylenolide I (ATLI), icariin (ICA), quercitrin (QUE), psoralen (PRN), kaempferol (KMP), ferulic acid (FA), protocatechuic acid (PCA), icaritin (ICT, an active pharmaceutical ingredient of ICA)	Tg (cmlc2: GFP) 24 hpf zebrafish embryos ISO (1 mM) for 48 h QXY extract (100, 200 and 600 µg/mL) for 48 h Propranolol (PRO, 10 µM) with ISO (1 mM) as the positive control	QXY (PRN, KMP, ICT) prevent ISO-induced cardiac hypertrophy and dysfunction in zebrafish QXY extract significantly decreased the cardiac output (CO), stroke volume, ejection fraction (EF), fractional shortening (FS), ventricular end-diastolic area (SVED), ventricular end-systolic area (SVES), SVED - SVES (ΔS), ventricular end-diastolic volume (VVED) and ventricular end- systolic volume (VVES)	Zhou et al. (2024b)
*Rhus chinensis* Mill. [Anacardiaceae*; Rhus chinensis radix*]	Eight novel Dammarane-type triterpenes (1–8) and a related known analogue (9)	Wild-type AB strain 2dpf zebrafish embryos 200 μM Verapami for 0.5 h Digoxin (0.8 μg/mL) as positive control	Dammarane-type triterpenes compound 5 in *Rhus chinensis* was the most active in preventing heart failure, showing better effects on increasing cardiac output and blood flow velocity	Ye et al. (2020)
Shen-Yuan- Dan Capsule (SYDC)	(Hirudo nipponia Whitman [Hirudinidae; Hirudo], *Astragalus membranaceus* (Fisch.) Bunge [Fabaceae; *Astragali* radix], *Codonopsis pilosula* (Franch.) Nannf. [Campanulaceae; *Codonopsis* radix], *Pheretima aspergillum* (E. Perrier) [Megascolecidae; *Pheretima*], *Eupolyphaga sinensis* Walker or *Steleophaga plancyi* (Bolívar) [Blattidae; *Eupolyphagae seu Steleophagae*], *Corydalis yanhusuo* W. T. Wang [Papaveraceae; *Corydalis* rhizoma], *Salvia miltiorrhiza* Bunge [Lamiaceae; *Salviae miltiorrhizae* radix et rhizoma], *Scrophularia ningpoensis* Hemsl. [Scrophulariaceae; *Scrophulariae* radix])	Wild-type AB strain 2dpf zebrafish embryos 200 mM Verapamil 30 min Digoxin (0.1 μg/mL) as positive control	SYDC improve cardiac output/blood flow; inhibite the increase of heart area and venous congestion ROS/MDA↓, SOD↑, Caspase-1↓, Caspase-3↓, Bax↓, IL-1β↓, NF-κB↓, TNF-α↓, Bcl-2↑	Li et al. (2021)
Notholaena rigida Davenp. [Pteridaceae; Notholaenae rigidae herba]/Betula humilis Schrank [Betulaceae; Betulae humilis folium]/Salvia barrelieri Etl. [Lamiaceae; Salviae barrelieri herba]	Novel pyxinol derivatives	Wild-type AB strain 48 hpf zebrafish embryos 200 μM Verapamil 30 min Enalapril (1 μg/mL) as positive control	Improve cardiovascular physiological indexes including heart beats, venous congestion, heart dilation, cardiac output, ejection fraction and fractional shortening	Liu et al. (2021b)
Black ginseng(*Panax ginseng* C. A. Mey. [Araliaceae; *Ginseng* radix nigra])	Ginsenoside Rg5	Wild-type AB strain 48 hpf zebrafish embryos 200 μM Verapamil 30 min Enalapril (1 μg/mL) as positive control	Rg5 can improve heart beats, venous congestion, heart dilation, cardiac output, ejection fraction and fractional shortening	Liu et al. (2021a)
Huoxin Pill (HXP)	*Ganoderma lucidum* (Curtis) P. Karst. [Polyporaceae; *Ganoderma*], *Panax ginseng* C. A. Mey. [Araliaceae; *Ginseng* radix et rhizoma], *Aconitum carmichaelii* Debeaux [Ranunculaceae; *Aconiti lateralis* radix praeparata], *Margaritifera margaritifera* (L.) [Unionidae; *Margaritiferae* concha], *Ursus arctos* L. [Ursidae; *Ursi fellis* pulvis], *Carthamus tinctorius* L. [Asteraceae; *Carthami tinctorii* flos], *Bufo bufo gargarizans* Cantor [Bufonidae; *Bufonis* venenum], *Moschus moschiferus* L. [Moschidae; *Moschus*], *Bos taurus domesticus* Gmelin [Bovidae; *Bovis calculi* artifactus] *and Borneolum syntheticum*	Wild-type AB strain,Tg (mpx:EGFP),Tg (myl7:EGFP) and Tg (nfkb:EGFP) 52 hpf zebrafish embryos 100 μM Verapamil 30 min	HXP can improve heart failure by increasing heart rate, reducing venous congestion, reducing pericardial cyst area, inhibiting apoptosis of zebrafish embryonic heart cells, reducing cardiac inflammation, and inhibiting oxidative stress HXP can reduce the level of NF-kB. gpx-1a↑, gss↑, hsp70↓, tnf-α↓, il-6↓, lck↓, apaf1↓, puma↓, caspase9↓	Li et al. (2024c)
Fangjihuangqi Decoction (FJHQD)	*Stephania tetrandra* S. Moore [Menispermaceae; *Stephaniae tetrandrae* radix] (STR) *Astragalus mongholicus* Bunge [Fabaceae; *Astragali* radix] (AR) *Atractylodes macrocephala* Koidz. [Asteraceae; *Atractylodis macrocephalae* rhizoma] (AMR) *Glycyrrhiza uralensis* Fisch. ex DC. [Fabaceae; *Glycyrrhizae* radix et rhizoma] (GRR)	Wild-type AB strain and Tg (cmlc2: eGFP) 76 hpf zebrafish larvae 200 μM Verapamil 30 min Digoxin (10 μM) as positive control	Improve cardiac function in zebrafish by regulating oxidative stress, inflammatory response and apoptosis-related pathways STR, AR and GRR, as well as A-STR (total Alkaloids from STR), F-AR (total Flavonoids from AR) and F-GRR (total Flavonoids from GRR) also have excellent anti-heart failure activity. nppa↓, il1b↓, il6↓, tnfa↓, caspase 1↓, caspase3↓	Li et al. (2022)
*Panax quinquefolius* L. [Araliaceae; *Panacis quinquefolii* radix] (AG)	Ginsenoside Rg3 (Rg3), Ginsenoside Rg5 (Rg5), Ginsenoside Rg6 (Rg6), malic acid, quinic acid, L-argininosuccinic acid, 3-methyl-3-butenyl-apinosyl (1→6) glucoside, pseudoginsenoside F11 (F11), and annonaine	Wild-type AB strain 48 hpf zebrafish embryos 200 μM Verapamil hydrohloride 1 h Digoxin (10 μg/mL) as positive control	Different locations produced AG with different anti-heart failure properties Rg3, Rg5, Rg6, malic acid, quinic acid, F11 were the pharmacodynamic markers responsible for anti-heart failure Rg3: ↓FGF1, STAT3; Rg5: ↓FGF2; Rg6: ↓FGF2, VEGFA; F11: ↓FGF1, VEGFA, FGF2	Dong et al. (2022)
Renshen-Fuzi herbal pair	*Panax ginseng* C. A. Mey. [Araliaceae; *Ginseng* radix et rhizoma] and *Aconitum carmichaelii* Debeaux [Ranunculaceae; *Aconiti lateralis* radix praeparata]	Wild-type AB strain 2dpf zebrafish embryos 200 μM Verapamil hydrochloride 30 min Digoxin (0.8 μg/mL) as positive control	Renshen-Fuzi with 1:2 ratio exhibited the best effect based on improving the cardiac function of heart failure zebrafish	Li et al. (2023)
Naoxintong (NXT) (*Astragalus mongholicus* Bunge [Fabaceae; *Astragali* radix], *Salvia miltiorrhiza* Bunge [Lamiaceae; *Salviae miltiorrhizae* radix et rhizoma], *Paeonia lactiflora* Pall. or *Paeonia veitchii* Lynch [Paeoniaceae; *Paeoniae rubra* radix], *Angelica sinensis* (Oliv.) Diels [Apiaceae; *Angelicae sinensis* radix], *Cinnamomum cassia* (L.) J. Presl [Lauraceae; *Cinnamomi* ramulus], *Prunus persica* (L.) Batsch [Rosaceae; *Persicae* semen], *Ligusticum chuanxiong* Hort. [Apiaceae; *Chuanxiong* rhizoma], *Spatholobus suberectus* Dunn [Fabaceae; *Spatholobi* caulis], *Morus alba* L. [Moraceae; *Mori* ramulus], *Carthamus tinctorius* L. [Asteraceae; *Carthami tinctorii* flos], *Achyranthes bidentata* Blume [Amaranthaceae; *Achyranthis* radix], *Boswellia sacra* Flueck. [Burseraceae; *Olibanum*], *Commiphora myrrha* (Nees) Engl. [Burseraceae; *Myrrha*], *Pheretima aspergillum* (E. Perrier) [Megascolecidae; *Pheretima*], *Buthus martensii* Karsch [Buthidae; *Scorpio*], *Hirudo nipponia* Whitman [Hirudinidae; *Hirudo*]	Paeoniflorin (PF), salvianolic acid B (Sal B)	Wild-type AB strain, Tg (cmlc2: eGFP), Tg (flk1: eGFP), Tg (heg1^Δ25^; cmlc2: eGFP), Tg (heg1^Δ25^; flk1: eGFP) 48 hpf zebrafish embryos 15 µM Terfenadine(TFD) for 48 h	Restored cardiac morphology (reduced pericardial edema, SV-BA distance), improved blood flow and heart rate; regulated HEG1-CCM signaling and myocardial genes (heg1, ccm2, myh6, myh7) The key bioactive components of cardiac protection in NXT are PF and Sal B	Hu et al. (2021)
Mountain-cultivated *Panax ginseng* C.A.Mey. [Araliaceae; *Ginseng radix et rhizoma*] (MCG)	A novel glycopeptide (APMCG-1)	Tg (kdrl:EGFP) 5dpf zebrafish larvae 10% high-fat diet for 8 h a day 3% glucose solution for 16 h a day, for 4 consecutive days	APMCG-1 increased the heart rate of zebrafish, reduced mortality, and restored blood flow velocity APMCG-1 reduced blood glucose, blood lipid levels, as well as myocardial injury and oxidative stress markers in zebrafish APMCG-1 increased the level of antioxidant enzymes and decreased the level of inflammatory factors. p-AKT↑, GRP78↓, p-PERKA↓, p-eIF2αA↓, ATF4A↓ CHOPA↓, Bax↓, Cleaved-Caspase3↓, Bcl2↑	Li et al. (2025)

This summary provides information on the regulatory effects of drugs or natural products on heart failure, using wild-type and transgenic zebrafish as experimental models. “↑” or “↓” illustrates the regulatory effects of drugs or natural products on different indicators. “↑” indicates increase or upregulation; “↓” indicates reduce or downregulation. dpf: days post-fertilization; hpf: hours post-fertilization. SV: sinus vein, BA: arterial bulb.

Studies demonstrate that dammarane-type triterpenes isolated from *Rhus chinensis* Mill. [Anacardiaceae*; R. chinensis radix*] roots increase cardiac output, blood flow velocity, and reduce pericardial edema and cardiomyocyte apoptosis in zebrafish. Subsequent investigations further isolated other dammarane-type triterpenoids from the same plant source, confirming their cardioprotective effects in zebrafish models (Ye et al., 2020; Ye et al., 2023). Ginsenosides Rg3, Rg5, Rg6, malic acid, quinic acid, and pseudoginsenoside F11 from *Panax quinquefolius* L. [Araliaceae; *Panacis quinquefolii radix*] exhibit differential effects on verapamil hydrochloride-induced HF in zebrafish. Specifically, Rg3, Rg6, quinic acid, and pseudoginsenoside F11 relieve pericardial dilation, venous congestion and abnormal SV-BA interval, while Rg5 and malic acid mainly reduce venous congestion (Dong et al., 2022). The cardioprotective effect of Rg5 may be due to its regulation of various metabolic pathways, including arachidonic acid metabolism, D-glutamate metabolism, phenylalanine metabolism, tricarboxylic acid cycle, glycerophospholipid metabolism, purine metabolism, steroid biosynthesis, and linoleic acid metabolism (Liu et al., 2021a). Moreover, a novel glycopeptide (APMCG-1) extracted from mountain-cultivated *P. ginseng* C.A.Mey. [Araliaceae; *Ginseng radix* et rhizoma] can increase zebrafish heart rate, restore blood flow velocity, reduce blood glucose and lipid levels, alleviate myocardial injury and oxidative stress, while increasing antioxidant enzyme levels, decreasing inflammatory factors, and mitigating HF in type 2 diabetic zebrafish (Li et al., 2025). The zebrafish HF model is also employed for screening Chinese medicine formulations. For example, *P. ginseng* C.A.Mey. [Araliaceae; *Ginseng* radix et rhizoma] and *Aconitum carmichaelii* Debeaux [Ranunculaceae; *Aconiti* lateralis radix praeparata], in a 1:2 dosage ratio, significantly increase cardiac output and blood flow velocity, while effectively inhibiting pericardial dilatation and venous congestion (Li et al., 2023). This paradigm now extends to a broader range of natural products and TCM formula research (Table 3). Examples include *Shen-Yuan-Dan Capsule*, *Huoxin Pill* (Li et al., 2024c), *Fangjihuangqi Decoction* (Li et al., 2022b), and *Naoxintong Decoction* (Hu et al., 2021). These formulations have been shown to improve HF by enhancing cardiac function, and their mechanisms of action have been scientifically validated. For instance, *Shen-Yuan-Dan Capsule* ameliorates verapamil-induced cardiac dysfunction by modulating oxidative stress and inflammatory markers such as MDA, ROS, SOD, Caspase-1, Bax, and IL-1β (Li et al., 2021).

Current zebrafish heart failure research relies largely on verapamil-induced models, which are convenient, rapid, and reproducible. However, this model mainly reflects calcium channel blockade and does not capture the multifactorial causes of human heart failure, limiting its translational relevance. Future studies should use diverse models, such as genetic cardiomyopathy models and ischemia-reperfusion injury (Zou et al., 2019), to better mimic human disease and provide a more comprehensive evaluation of therapeutics.

### 2.4 Angiogenesis model

Angiogenesis involves the growth and formation of new vascular networks from pre-existing vessels, regulated by VEGF/VEGFR signaling (Ramjiawan et al., 2017), and includes processes such as sprouting, endothelial cell migration, proliferation, and lumen formation. Zebrafish have become a widely used model for vascular research due to their genetic similarity to humans and embryonic transparency (Hung et al., 2012). Commonly used transgenic strains, such as Tg (fli1a:EGFP) and Tg (Flk1:GFP) label vascular endothelial cells with green fluorescent protein, enabling real-time visualization of vascular dynamics. Pharmaceutical regulatory effects on zebrafish vasculature are assessed using subintestinal vessels (SIVs) sprouting assays and intersegmental vessels (ISVs) damage tests (Fan et al., 2017). Vascular injury models are induced using compounds such as SU5402 (FGFR antagonist), PTK787 (VEGFR2 inhibitor), and vascular endothelial growth factor (VEGF) receptor tyrosine kinase inhibitor II (VRI) (Tal et al., 2014; Zhang et al., 2014). In zebrafish embryonic development, angiogenesis begins 20 hpf (Hong et al., 2008) with its activity peaking between 24 and 72 hpf. Angiogenesis can be assessed by counting fluorescently tagged ISVs (Camus et al., 2012).

First, in TCM, botanicals and natural substances promote zebrafish SIVs production, correct ISVs deficits and other phenotypic improvements, and modulate angiogenesis-related targets or pathways to treat angiogenic diseases (Li et al., 2022a). The main process involves VEGF, PI3K-Akt, and MAPK. As shown in Table 4, *Dalbergia odorifera* T.C.Chen [Fabaceae; *Dalbergiae odoriferae* lignum] extract upregulates vascular endothelial growth factor receptors (VEGFRs, including kdr, kdrl, and flt-1) to repair VRI-induced ISVs damage in zebrafish (Fan et al., 2017). Litospermic acid, the active metabolite of *S. miltiorrhiza* Bunge [Lamiaceae; *Salviae miltiorrhizae* radix et rhizoma] Bge (Liang et al., 2024), and notoginseng saponins (Hong et al., 2008), promote angiogenesis by regulating VEGF-KDR/Flk-1 and VEGF, PI3K-Akt signaling pathways, respectively. Notably, different Chinese botanical medicine ingredients have diverse characteristics. For example, Ilexsaponin A1, the main ingredient of *Ilex pubescens* Hook. and Arn. [Aquifoliaceae; *I. pubescens* radix], modulates Akt/mTOR, MAPK/ERK and Src-FAK signaling pathways to reduce VRI-induced vascular dysfunction (Li J. J. et al., 2017), while *Perilla frutescens* (L.) Britton [Lamiaceae; *Perillae frutescentis* folium] volatile oil and perillaldehyde activate the p-ERK1/2/ERK1/2 pathway and upregulate the Bcl-2/Bax ratio to increase angiogenesis in a sunitinib-induced zebrafish vascular damage model (Zhou et al., 2021). Many studies have revealed that *Gastrodia elata* Blume [Orchidaceae; *Gastrodiae* rhizoma] and its polysaccharide and non-polysaccharide metabolites induce angiogenesis in a dose-dependent manner (Liu et al., 2020). *Achyranthes bidentata* Blume [Amaranthaceae; *Achyranthis bidentatae* radix] and *Cyathula officinalis* K.C.Kuan [Amaranthaceae; *Cyathulae* radix] extracts increase endothelial cell migration and SIVs sprouting (Zhou et al., 2017), while *Carthamus tinctorius* L. [Asteraceae; *Carthami tinctorii* flos] (Honghua) extracts exert pro-angiogenic effects via upregulating vascular development-associated genes like IGF1, NRP2, and VEGFR3 (Zhou et al., 2014). Aucubin from *Eucommia ulmoides* Oliv. [Eucommiaceae; *Eucommiae* cortex] can alleviate VRI-induced suppression of vascular-related genes (flt-1, kdrl, ang-1/2, etc.) in zebrafish embryos and restore ISVs integrity (He et al., 2023). Additionally, other natural products, including ferulic acid, curculigoside, deoxycholic acid, ursodeoxycholic acid, 1-beta-hydroxyalantolactone, and cinobufotalin, have also been shown to promote angiogenesis (Zhu et al., 2023).

**TABLE 4:** Evaluation of natural products for angiogenic and anti-angiogenic effects using zebrafish models.

Bioactivity	Source	Extracts/Compounds	Model	Finding	Reference
Anti-Angiogenic Effects	Wild Chrysanthemum (*Chrysanthemum indicum* L. [Asteraceae; *Chrysanthemi indici flos*])	Wild chrysanthemum water extract (WCWE)	Tg (fli1a-EGFP) 22hpf zebrafish embryos WCWE (200 μg/mL) for 26 h PTK 787 (5 μg/mL) as positive control	Significant anti-angiogenic effect via proteasome/β-catenin signaling downregulation	Tu et al. (2021)
	Red ginseng(*Panax ginseng* C. A. Mey. [Araliaceae; *Ginseng* radix rubra])	Ginsenoside Rh2(G-Rh2)	Tg (fli1: EGFP) 24hpf zebrafish embryos G-Rh2 (84.85 μM) for 24 h SU5416 (5 μM) as positive control	G- Rh 2 inhibited intersegmental angiogenesis in a dose-dependent manner in the range of 30–90 μM The optimal concentration of G-Rh2 was 84.85 μM, and the concentration of 120 μM or more was toxic to zebrafish embryos G-Rh 2 may exert anti-angiogenic activity by down-regulating VEGF in zebrafish embryos	Ma et al. (2020)
	*Andrographis paniculata* (Burm.f.) Nees [Acanthaceae; *Andrographidis* herba]	Andrographolide derivative (AGP-40)	Tg (fli-1: EGFP) 24hpf zebrafish embryos AGP-40(30 μM) for 12 h VEGFR tyrosine kinase inhibitor II (VRI) (200 nM) as a positive control	AGP-40 antiangiogenic activity at 10–30 μM Toxicity observed from 8 hpt; mortality by 24 hpt Anti-angiogenic via PI3K/Akt and MEK/ERK pathways	Li et al. (2016)
	Erxian Decoction (EXD)	*Epimedium sagittatum* (Sieb. et Zucc.) Maxim. [Berberidaceae; *Epimedii* folium], *Curculigo orchioides* Gaertn. [Hypoxidaceae; *Curculiginis* rhizoma], *Morinda officinalis* How [Rubiaceae; *Morindae officinalis* radix], *Angelica sinensis* (Oliv.) Diels [Apiaceae; *Angelicae sinensis* radix], *Phellodendron chinense* C. K. Schneid. [Rutaceae; *Phellodendri chinensis* cortex], *Anemarrhena asphodeloides* Bunge [Asparagaceae; *Anemarrhenae* rhizoma]	Tg (fli1a: EGFP)^y1^ 24hpf zebrafish embryos EXD(1 mg/mL) for 24 h, 48 h SU5416(4 μM) as positive control	Most of the intersegmental vessels (ISVs) failed to form when treated with 1 mg/mL EXD for 48 h The subintestinal vessel plexus (SIVs) almost regressed when treated with 1 mg/mL EXD for 72 h EXD reduced VEGF-A mRNA expression and hif-1α protein levels	Yu et al. (2012)
	*Picrasma quassioides* (D. Don) Benn. [Simaroubaceae; *Picrasmae quassioidis* lignum]	1-methoxycarbony-β-carboline (MCC)	Tg (fli1: EGFP) 24hpf zebrafish embryos, adult zebrafish MCC (50 μM) SU5416(2.5 μM) as positive control	MCC can inhibit the regeneration of caudal fin vessels after injury Dose-dependent ISV inhibition (48 hpf); SIV regression at 72 hpf ANG↓, EGF↓, bFGF↓, GRO↓, IGF-1↓, PLG↓ MMP-1↓	Lin et al. (2018)
	*Reynoutria japonica* Houtt. [Polygonaceae; *Polygoni cuspidati* rhizoma et radix] (PCRR)	The Extract of PCRR	Wild-type AB strain 24hpf zebrafish embryos VEGF (10 ng/mL) and PCRR(3,10,30,100 μg/mL) for 48 h 200 μg/mL Avastin as positive control	PCRR extract suppressed the formation of sub-intestinal vessels in zebrafish embryos	Hu et al. (2018)
	*Anemarrhena asphodeloides* Bunge [Asparagaceae; *Anemarrhenae* rhizoma]	Timosaponin AIII (Timo AIII)	Tg (fli-1a: EGFP)^y1^ 24hpf zebrafish embryos ISVs: Timo AIII (0.5, 1, 2, and 3 µM) for 12 h SIVs: Timo AIII (0.5, 1, and 2 µM) for 48 h	Timo AIII decrease ISVs (dose-dependent) Timo AIII inhibits SIVs growth and reduce total SIVs area Antiangiogenic via VEGF/PI3K/AKT/MAPK pathway	Zhou et al. (2019b)
	*Curcuma wenyujin* Y. H. Chen et C. Ling [Zingiberaceae; *Curcumae* rhizoma]	Furanodiene	Tg (fli1: EGFP) 48hpf zebrafish embryos Furanodiene (50 M) for 72 h SU5416(5 μM) as positive control	Furadiene exposure inhibited endothelial cell growth, invasion, migration and tube formation by modulating PI 3K pathway, showing potential anti-angiogenic effects	Zhong et al. (2012)
	*Rheum palmatum* L. [Polygonaceae; *Rhei* radix et rhizoma]	Rhein	Tg (fli1a: EGFP)^y1^ 24hpf zebrafish embryos Rhein (20 μM) for 48 h or 72 h	Rhein ↓ ISV formation (complete blockade at 48 and 72 hpf); ↓ SIVs formation (complete inhibition at 72 hpf) Antiangiogenic via regulation of angpt2/tie2 expression	He et al. (2011)
	*Dysosma versipellis* (Hance) M. Cheng [Berberidaceae; *Dysosmae* rhizoma]	Kaempferol	Tg (kdrl:GRCFP)^zn1^ 6hpf zebrafish embryos Kaempferol 40 μM	Kaempferol ↓ ISV growth (zebrafish embryos; specific inhibition) Kaempferol + FGFR inhibitor can complete ISV growth blockade Antiangiogenic via VEGF/FGF pathways; VEGF receptor ↓	Liang et al. (2015)
	*Paris polyphylla* Sm. [Melanthiaceae; *Paridis* rhizoma]	Polyphyllin D (PD)	Tg (flil:EGFP) 20hpf zebrafish embryos Polyphyllin D (PD) (0.156 μM, 0.313 μM) for 2 h SU5416(2 μM) as positive control	PD (0.156–0.313 μM) ↓ ISVs formation in zebrafish embryos (dose-dependent) PD did not significantly impact the general development of zebrafish, but particularly hindered angiogenesis	Chan et al. (2011)
	*Chrysanthemum indicum* L. [Asteraceae; *Chrysanthemi indici flos*] and *Commiphora myrrha* (Nees) Engl. [Burseraceae; *Commiphorae myrrhae radix*]	Wild Chrysanthemum water extract (WCWE). Uniflower Swisscentaury Root water extract (USRWE)	Tg (Fli1a-EGFP) 22hpf zebrafish embryos Heat-clearing and detoxicating TCM (200 μg/mL) for 26 h PTK 787(5 μg/mL) as positive control	Anti-angiogenic effects: Dual modulation of pro-angiogenic/negative regulators WCWE: VEGF-A↑, VEGFR-2↑, VEGFR-1↑, Dll4↑, Notch1a↑, hey2↑, efnb2a↑, COX2↓, mmp9, mmp2↓ USRWE: VEGF-A↑, VEGFR-2↑, VEGFR-1↑, Dll4↑, hey2↑, efnb2a↑, COX2 ↓	Tu et al. (2016)
	Polymethoxylated Flavonoid from Citrus	Nobiletin	Tg (fli1: EGFP) 24hpf zebrafish embryos Nobiletin (10μM, 30μM, 100 μM) for 8 h VEGFR inhibitor II (0.2 mM) served as positive control	Anti-angiogenic by VEGF-A pathway and cell cycle-related proteins modulation; inhibited ISVs at 30–100 μM	Lam et al. (2011)
	A bioactive flavonoid present in many Chinese herbal medicine	Quercetin	Tg (fli1: EGFP) 6hpf zebrafish embryos Quercetin (50,100,200 μM) treatment continued to 72hpf	Quercetin is toxic to zebrafish embryos at higher concentrations (≥300 μM) Quercetin has anti-angiogenic activity. Quercetin is involved in inhibiting extracellular signal-regulated kinase signaling pathway *in vivo* and *in vitro*	Zhao et al. (2014)
	*Euphorbia pekinensis* Rupr. [Euphorbiaceae; *Euphorbiae pekinensis* radix]	The water extract of *Euphorbia pekinensis* Rupr.(EP) vinegar preparation (WEVEP)	Tg (flk: mCherry) 6hpf zebrafish embryos WEVEP from 100 µg/mL to 250 µg/mL treatment continued to 72hpf PTK787 2HCl (100 ng/mL) as the positive control	LD_50_ (WEVEP) = 250μg/m-300 μg/mL Angiogenesis was inhibited by the WEVEP in zebrafish (from 100 µg/mL to 250 µg/mL) WEVEP: Met↓, IGF1↓, CTGF↓, VEGFA↓, VEGFR3↑	Zhang et al. (2017)
	*Angelica sinensis* (Oliv.) Diels [Apiaceae; *Angelicae sinensis* radix]	n-butylidenephthalide (BP)	Wild-type AB strain At the 13somite stage (ss) zebrafish embryos (0.01 µg/mL) BP treatment from at the 13 (ss) to 72hpf Stained for endogenous alkaline phosphatase (AP) activity	BP could impairs SIVs in zebrafish BP have anti-angiogenic effects, which are related to the activation of p38 and ERK 1/2, but not to SAPK/JNK and Akt signaling pathways. The mechanism is to inhibit cell cycle progression and induce apoptosis	Yeh et al. (2011)
	*Euphorbia pekinensis* Rupr. [Euphorbiaceae; *Euphorbiae pekinensis* radix]. (EP)	*Euphorbia pekinensis* (EP) water extract, active ingredient composition (AIC) containing 3,3′-O-dimethoxy ellagic acid, quercetin, and ingenol	Tg (flk1: mCherry) 6hpf Zebrafish embryos EP water extract solution (200 μg/mL) treatment from 6hpf to 72hpf PTK787 2HCl (100 ng/mL) as a positive control	EP water extract (100–250 μg/mL) inhibited angiogenesis (concentration-dependent) AIC (equivalent active ingredient dose to EP): Significant anti-angiogenic activity 3,3′-O-dimethoxy ellagic acid, quercetin, ingenol: antiangiogenic Oleanolic acid, euphol: inactive	Zhang et al. (2022a)
	The roots, rhizomes, and stems of plants belonging to the *Papaveraceae*, *Ranunculaceae*, and *Euphorbiaceae*	Berberine (BBR)	Wild-type AB strain 1hpf Zebrafish embryos BBR (0.01–0.75 mM) treatment from 1hpf to 72hpf	LC_50_ of BBR = 0.660 ± 0.062 (mM/L ± SEM) 0.5 mM BBR slightly reduced ISVs; 0.75 mM BBR reduced dorsal aorta (DA) and ISVs ↓eNOS/VEGF-dependent pathway gene: VEGF, VEGFR2, NRP1 a, HIF-1α, Nos2 a, Nos2 b, COX-2 a and COX-2 b	Nathan et al. (2023)
	Citrus spp.	Naringenin	Tg (fli1a: EGFP) 20 hpf zebrafish embryos Naringenin (3, 10, 30, 100 μM) for 24 h Pretreated with VRI (500 ng/mL) for 4 h	Naringenin dose-dependently decreased SIVs development, suggesting anti-angiogenic action	Chen et al. (2018a)
Promotes Angiogenesis Effects	Citrus spp.	Naringin	Tg (fli1a: EGFP) 20 hpf Zebrafish embryos Naringin (3, 10, 30, 100, 200 μM) for 24 h Pretreated with VRI (500 ng/mL) for 4 h.	Naringin restore vascular loss and increase the number of intact ISVs. flt1↑, kdr↑, kdrl↑, tie2↑	Chen et al. (2018a)
	*Panax notoginseng* (Burkill) F.H.Chen [Araliaceae*; Notoginseng radix et rhizoma*]	Saponin extract from *Panax notoginseng* (PNS)	Tg (fli1: EGFP) 1–4 cell stage Zebrafish embryos PNS (100–300 μg/mL) for 72–120 h VEGF (1 ng/mL) as positive control	PNS-treated zebrafish embryo has longer and more vascular branches from the subintestinal vascular basket in SIVs PNS promotes angiogenesis via the VEGF-KDR/Flk-1 and PI3K-Akt-eNOS pathways	Hong et al. (2008)
	Danshen and Gegen Decoction (DG) (*Salvia miltiorrhiza* Bunge [Lamiaceae, *Salviae miltiorrhizae* radix et rhizoma]	Danshen and Gegen	Tg (fli1: EGFP)^y1^/+(AB) 2-cell stage Zebrafish embryos DG (0,125, 250, 500 μg/mL) for 96 h	DG exhibited significant angiogenic effects in zebrafish embryos With the increase of DG concentration, the germination of subintestinal vessels (SIVs) of zebrafish embryos was enhanced	Hu et al. (2013)
	Yiqi Fumai lyophilization injection (YQFM) (*Panax ginseng* C. A. Mey. [Araliaceae; *Ginseng* radix et rhizoma];, *Ophiopogon japonicus* (Thunb.) Ker Gawl. [Asparagaceae; *Ophiopogonis* radix];, *Schisandra chinensis* (Turcz.) Baill. [Schisandraceae; *Schisandrae chinensis* fructus].)	Ginsenoside Rb2	The model groups were given simvastatin solution (1 mg/mL) The administration groups were given 12.5, 25, 50, 100 and 200.0 mg/mL YQFM Astragaloside IV (23.5 μg/mL) as positive control	Ginsenoside Rb2 (YQFM): Promote angiogenesis YQFM (200 mg/mL): More significant angiogenesis promotion	Xu et al. (2023)
	*Dalbergia odorifera* T. C. Chen [Fabaceae; *Dalbergiae odoriferae* lignum]	*Dalbergia odorifera* extract(DOE)	Tg (fli1a-EGFP)^y1^ 24hpf zebrafish embryos 80 nM VEGF receptor kinase inhibitor II (VRI) DOE (1–10 μg/mL) for 48 h	Restored VRI-induced ISVs and SIVs damage via VEGFRs/PI3K/MAPK pathways (dose-dependent)	Fan et al. (2017)
	*Typha angustifolia* L. or *Typha orientalis* C. Presl [Typhaceae; *Typhae* pollen] (TP)	*Typhae Pollen* (TP), Carbonized *Typhae Pollen* (CTP)	Tg (fli-1: EGFP)^y1^ 24 hpf zebrafish embryos 500 ng/mL VRI for 3 h TP and CTP (6.25, 12.5, 25, 50, and 100 μg/mL)	TP and CTP can dose-dependently restore VRI-induced ISVs defects and promote angiogenesis. flt1↑, kdr↑, kdrl↑	Gao et al. (2021)
	*Codonopsis pilosula* (Franch.) Nannf. [Campanulaceae; *Codonopsis* radix]	Lobetyolin (LBT)	Tg (kdrl: mCherry) 6hpf Zebrafish embryos	LBT can effectively promote angiogenesis, lead to vascular morphological abnormalities, have low toxicity, and significantly promote neuronal development in zebrafish	Wang et al. (2020a)
	*Angelica sinensis* (Oliv.) Diels [Apiaceae; *Angelicae sinensis* radix], *Ligusticum chuanxiong* Hort. [Apiaceae; *Chuanxiong* rhizoma], Panax ginseng C. A. Mey. [Araliaceae; Ginseng radix et rhizoma], etc.	Ferulic acid(FA)ˎ Curculigostide(CUR)ˎDeoxycholic acid (DA)ˎUrsodeoxycholic acid (UA)ˎ1-beta-hydroxyalantolactone (BHA)ˎCinobufotalin (CIN)ˎGinsenosides Rb3ˎRc	Tg (vegfr2: GFP) 24hpf zebrafish embryos Echorionated with 1 mg/mL of Pronase E before treatment Rg1(40 μM) as positive control	FA: promoted angiogenesis in ISVs (40–160 μM; dose-dependent) CUR: promoted angiogenesis in ISVs (160 μM) DA, UA, BHA, CIN, Rb3, Rc: Pro-angiogenic effects	Zhu et al. (2023)
	Compound Danshen Dripping Pill (CDDP)	Mixture of extracts from *Salvia miltiorrhiza* Bunge [Lamiaceae*; Salviae miltiorrhizae* radix et rhizoma], *Panax notoginseng* (Burkill) F.H.Chen [Araliaceae*; Notoginseng* radix et rhizoma], and other herbs	Tg (fli1: EGFP) 24hpf zebrafish embryos CDDP (0.3, 0.6, 0.9 and 1.2 mg/mL) for 48 h Pretreated with VRI (100 nM) for 24 h, CDDP for another 24 h	CDDP dose-dependently enhanced angiogenesis in SIVs and optic arteries (OAs) and alleviated ISVs deficits via the VEGF/VEGFR and PI3K/AKT signaling pathways	Hu et al. (2022)
	*Rehmannia glutinosa* (Gaertn.) DC. [Orobanchaceae; *Rehmanniae* radix] (RR)	RR sub-fraction C1-1	Tg (fli1: EGFP)^y1^/+ (AB) 1–4 cell stage zebrafish embryos for 72 h–96 h Vascular endothelial growth factor (VEGF) as positive control	Sub-fraction C1-1 (190–780 ng/mL) promotes angiogenesis in zebrafish embryos, as evidenced by a significant increase in the number of ectopic sprouts C1-1 (780 ng/mL): MMP-9↑, ANGPT1↑, EGFR↑, EPHB4↑, Ephrin B2↓, Flt-1↓, Ets-1↓	Liu et al. (2014)
	Shuxinyin formula(SXY)	*Codonopsis pilosula* (Franch.) Nannf. [Campanulaceae; *Codonopsis* radix], *Astragalus membranaceus* (Fisch.) Bunge [Fabaceae; *Astragali* radix], *Rehmannia glutinosa* (Gaertn.) DC. [Orobanchaceae; *Rehmanniae* radix], *Ophiopogon japonicus* (Thunb.) Ker Gawl. [Asparagaceae; *Ophiopogonis* radix], *Lycium barbarum* L. [Solanaceae; *Lycii* fructus], *Taxillus sutchuenensis* (Lecomte) Danser [Loranthaceae; *Taxilli* herba], *Pueraria lobata* (Willd.) Ohwi [Fabaceae; *Puerariae lobatae* radix]	Tg (fli1a: EGFP)^y1^ 1dpf zebrafish embryos were dechorionated SXY (10, 30, 100, 200, 400 µg/mL) for 48 h SXY and VRI (160 nM) for 24 h VEGF (100 ng/mL) as positive control	SXY facilitated angiogenesis via VEGF/PI3K/Akt/MAPK signaling pathways, cellular junctions, apoptosis, and autophagy	Zhou et al. (2019a)
	Guanxinning tablets (GXNT) (*Salvia miltiorrhiza* Bunge [Lamiaceae; Salviae miltiorrhizae radix et rhizoma] and *Ligusticum striatum* DC. [Apiaceae; Ligustici striati rhizoma])	Salvianolic acid B and ferulic acid	Tg (Fli:EGFP) 24 hpf zebrafish embryos VEGF (100 nM) was used as positive control	Salvianolic acid B and ferulic acid synergistically promote angiogenesis in HUVECs and zebrafish Promoted endothelial proliferation and cell cycle progression ↑multiple VEGF receptors and ligands	Chen et al. (2022)
	*Carthamus tinctorius* L. [Asteraceae; *Carthami tinctorii* flos]	Carthami Flos(CF) whole extract	Tg (fli1: EGFP)^y1^/+ (AB) 72hpf zebrafish larvae Rg1 (20 µM) as positive control	Carthami Flos (CF) extract can increase the number of vascular sprouting in zebrafish, and has a significant pro-angiogenic effect ↑genes for angiogenesis and associated growth factors and receptors: IGF1, CTGF, NRP2, VEGFR3, HIF1A, MMP2, MMP9, TIMP2, PLG, PLAU, ITGAV, ITGB3, beta-catenin, PECAM1, ANGPT1, TIE-2, PDGFR-B, CDH5, S1PR1, FGF2, Shh, TGFRB1, and Ephrin B2	Zhou et al. (2014)
	Xinkeshu tablets	*Salvia miltiorrhiza* Bunge [Lamiaceae; *Salviae miltiorrhizae* radix et rhizoma], *Pueraria lobata* (Willd.) Ohwi [Fabaceae; *Puerariae lobatae* radix], *Panax notoginseng* (Burkill) F. H. Chen [Araliaceae; *Notoginseng* radix et rhizoma], *Crataegus pinnatifida* Bunge [Rosaceae; *Crataegi* fructus], *Aucklandia lappa* Decne [Asteraceae; *Aucklandiae* radix]	Tg (fli1a: EGFP) 20 hpf zebrafish embryos 0.175 μg/mL PTK787 for 24 h 40 ng/mL VEGF as positive control	XKS methanol extract composition: 116 components (phenolic acids, isoflavones, triterpenes) ↑ ISV and SIV growth (zebrafish embryos) Multi-pathway activation: PPAR, AGE-RAGE, NOD-like receptor, VEGF, PI3K/Akt, metabolic pathways	Liu et al. (2023)
	*Achyranthes bidentata* Blume [Amaranthaceae; *Achyranthis* radix] (RAB) and *Cyathula officinalis* K. C. Kuan [Amaranthaceae; *Cyathulae* radix] (RC)	Whole extracts of RAB and RC.	Tg (fli1: EGFP)^y1^/+(AB) 1–4 cell stage zebrafish embryos RAB and RC whole extracts (0–200 µg/mL)	The extracts of RAB and RC significantly increased the number of sprouting of zebrafish SIVs Induced angiogenesis mainly by promoting endothelial cell migration	Zhou et al. (2017)
	*Ilex pubescens* Hook. et Arn. [Aquifoliaceae; *Ilicis pubescentis* radix et caulis]	Ilexsaponin A1	Tg (fli-1: EGFP) 24 hpf zebrafish embryos Pretreated 500 ng/mL VRI for 3 h Ilexsaponin A1 (1µM, 3µM, 10µM, 30 µM) for 24 or 48 h	Ilexsaponin A1 can improve the inhibition of VRI on zebrafish intersegmental blood vessels and subintestinal blood vessels Its role in promoting angiogenesis probably by activating Akt/mTOR, MAPK/ERK and Src-FAK-dependent signalling pathways	Li et al. (2017a)
	*Perilla frutescens* (L.) Britton [Lamiaceae; *Perillae* folium	Essential oil from P. *frutescens* (EOPF) and its main component perillaldehyde	Tg (flk1: EGFP) 10hpf zebrafish embryos Sunitinib (1.5 µg/mL) for 48hpf	EOPF and Perillaldehyde significantly promoted the formation of ISV and increased ISV index(dose-dependent) Activated p-ERK1/2/ERK1/2 pathway and upregulated Bcl-2/Bax ratio	Zhou et al. (2021)
	Tongnao Decoction (TND)	*Uncaria rhynchophylla* (Miq.) Miq. ex Havil. [Rubiaceae; *Uncariae ramulus cum uncis*], *Rhodiola crenulata* (Hook.f. and Thomson) H. Ohba [Crassulaceae; *Rhodiolae crenulatae* radix et rhizoma], *Ligusticum chuanxiong* Hort. [Apiaceae; *Chuanxiong* rhizoma], *Arisaema erubescens* (Wall.) Schott [Araceae; *Arisaematis* rhizoma], *Anemone altaica* Fisch. ex C. A. Mey. [Ranunculaceae; *Anemones altaicae* rhizoma], *Gastrodia elata* Blume [Orchidaceae; *Gastrodiae* rhizoma], *Bombyx batryticatus* (white stiff silkworm) [Bombycidae; *Bombyx batryticatus*], *Hirudo nipponia* Whitman [Hirudinidae; *Hirudo*]	Tg (fli-1: EGFP) 24 hpf zebrafish embryos VRI (575 nM) for 4 h	Toxicity thresholds: LC_50_ = 4782 µg/mL; MNLC = 1,375 µg/mL (1:10) TND rescues VRI-induced blood vessel defects (dose-dependent) Activates VEGFR-2, PI3K/Akt and Raf-MEK1/2-ERK1/2 pathways	Wang et al. (2020b)
	*Gastrodia elata* Blume [Orchidaceae; *Gastrodiae* rhizoma] (GR, Gastrodia elata)	GR and separation of the polysaccharide (RGP) and non-polysaccharide (NRGP)	Tg (vegfr2: GFP) 48hpf zebrafish embryos PTK787(0.2 µg/mL) Danhong Injection (DHI) as positive control	RGP and NRGP (100 μg/mL): Significant angiogenesis (concentration-dependent) Steaming may reduce NRGP pro-angiogenic effect	Liu et al. (2020)
	Diammonium glycyrrhizinate injection (DGI) (*Glycyrrhiza uralensis* Fisch. ex DC. [Fabaceae; *Glycyrrhizae* radix et rhizoma])	Glycyrrhizic acid (GA)	Tg (fli-1: EGFP) 24 hpf/72 hpf zebrafish embryos PTK787(0.2 µg/mL) Danhong Injection (DHI) as positive control	DGI dose-dependently (25–100 µg/mL) reversed PTK787-induced vascular injury, significantly increasing the length and number of ISVs and SIVs in zebrafish Activated mTOR/HIF-1 signaling pathway pik3cb↑, pdpk1↑, akt1↑, ikbka↑, mtor↑, rps6kb↑, hif1ab↑, vegfaa↑	Yu et al. (2025)
	*Eucommia ulmoides* Oliv. [Eucommiaceae; *Eucommiae* cortex]	Aucubin	Tg (fli1a-EGFP) + (AB) 24hpf zebrafish embryos VRI (500 ng/mL) for 3 h	Aucubin demonstrates a protective effect in zebrafish for the formation of intact ISVs via modulating VEGF-VEGFR and Ang-Tie signaling pathways. flt-1↑, kdr↑, kdrl↑, vegfaa↑, ang-1↑, ang-2↑, tie1↑, tie2↑	He et al. (2023)
	Fufang E ′jiao Jiang (FEJ)	*Equus asinus* L. [Equidae; *Asini corii* gelatina]; *Rehmannia glutinosa* (Gaertn.) DC. [Orobanchaceae; *Rehmanniae* radix praeparata]; *Panax ginseng* C. A. Mey. [Araliaceae; *Ginseng* radix rubra]; *Codonopsis pilosula* (Franch.) Nannf. [Campanulaceae; *Codonopsis* radix]; *Crataegus pinnatifida* Bunge [Rosaceae; *Crataegi* fructus]	Tg (flk1: EGFP), Tg (gata1: dsRed) and Wild-type AB strain 48hpf AB strain zebrafish PHZ (0.175ug/mL) 20hpf Tg (flk1: EGFP) PTK787(0.15 μg/mL)	Promote immune cell production, increase the number of red blood cells, activate complement and coagulation cascades, ECM receptor interaction, and PI3K-Akt pathway. c8a↑, c4b↑, c5↑, f2↑, itga9↑, gp9↑, gys2↑,rpsa↓	Li et al. (2024b)
	*Salvia miltiorrhiza* Bunge [Lamiaceae; *Salviae miltiorrhizae* radix et rhizoma], *Crocus sativus* L. [Iridaceae; *Croci* stigma]	Lithospermic acid (LA)	Tg (fli1a: EGFP) 20 hpf zebrafish embryos PTK787 (0.175 μg/mL) for 24 h LA (25, 20 and 100 μM) and PTK787 for 24 h	Luteolin (LA) restored the length of intersegmental vessels (ISVs) damaged by PTK787 and promoted the sprouting of subintestinal vessels (SIVs) in zebrafish in a concentration - dependent manner LA exhibits proangiogenic effects by regulating the VEGF, PI3K - Akt, and MAPK pathways. akt1↑, egfr↑, src↑, bcl - 2↑, erk1↑, mek1↑, igf1↑, hsp90aa1↑, mmp9↑, vegfa↑, akt2↑, rhoa↑, caspase3↓, caspase9↓, bax↓	Liang et al. (2024)
	Qilong capsule (QLC) (*Astragalus membranaceus* (Fisch.) Bunge [Fabaceae; *Astragali* radix], *Pheretima aspergillum* (E. Perrier) Tsai et al. [Megascolecidae; *Pheretimae*], *Salvia miltiorrhiza* Bunge [Lamiaceae; *Salviae miltiorrhizae* radix et rhizoma], *Angelica sinensis* (Oliv.) Diels [Apiaceae; *Angelicae sinensis* radix] and etc.	The extract of Qilong capsule (QLC)	Wild-type AB strain and Tg (fkl:EGFP) 20 hdf zebrafish embryos PTK787 to 44hpf Danhong injection (DHI) as positive control	QLC can significantly increase the number and length of intersegmental blood vessels in zebrafish	Lin et al. (2023)

This summary provides information on the regulatory effects of drugs or natural products on blood vessels, using wild-type and transgenic zebrafish as experimental models. “↑” or “↓” illustrates the regulatory effects of drugs or natural products on different indicators. “↑” indicates increase or upregulation; “↓” indicates reduce or downregulation. ISVs: intersegmental vessels, SIVs: subintestinal vein vessels; dpf: days post-fertilization; hpf: hours post-fertilization. MNLC: maximum nonlethal concentration, LC_50_: Lethal concentration 50%.

On the other hand, the equilibrium between anti-angiogenic and pro-angiogenic processes maintains physiological homeostasis (Sajib et al., 2018). Tumor malignancy is closely associated with pathological angiogenesis (Kondo et al., 2002; Mantovani, 2010), which makes angiogenesis inhibition a critical target for cancer therapy and anti-tumor drug development (Viallard and Larrivée, 2017). In addition to promoting angiogenesis, certain natural products exhibit anti-angiogenic effects. For instance, *Polygonum cuspidatum* Siebold and Zucc. [Polygonaceae; *Polygoni cuspidati* rhizoma et radix] extract inhibits angiogenesis by suppressing VEGF-induced endothelial cell migration and proliferation (Hu et al., 2018). Timosaponin AIII, isolated from *Anemarrhena asphodeloides* Bunge [Asparagaceae*; Anemarrhenae* rhizoma], suppresses angiogenesis by directly inhibiting the VEGF/PI3K/AKT/MAPK signaling cascade (Zhou et al., 2019b). Moreover, the andrographolide derivative AGP-40 disrupts vascularization by inhibiting PI3K/Akt and MEK/ERK pathways (Li et al., 2016), while furanodiene derived from *C. longa* L. [Zingiberaceae; *Curcumae longae* rhizoma] reduces endothelial cell growth, invasion, and angiogenesis via modulation of the PI3K pathway (Zhong et al., 2012). *Chrysanthemum indicum* L. [Asteraceae; *Chrysanthemi indici* flos] and *Commiphora myrrha* (Nees) Engl. [Burseraceae; *Commiphorae myrrhae* radix] inhibit angiogenesis by differentially regulating VEGFRs, the Notch pathway, Dll4, hey2, and matrix-degrading enzymes (MMP2/9, COX2) in zebrafish (Tu et al., 2016; Tu et al., 2021). Additionally, 1-methoxycarbony-β-carboline from *Picrasma quassioides* (D.Don) Benn. [Simaroubaceae; *Picrasmae* radix] suppresses angiogenesis by downregulating autocrine proteins such as ANG and bFGF (Lin et al., 2018). Furthermore, *Dysosma versipellis* (Hance) M. Cheng [Berberidaceae; *Dysosmae* rhizoma] (Liang et al., 2015), polyphyllin D (Chan et al., 2011), and nobiletin (Lam et al., 2011) inhibit angiogenesis by blocking VEGF/FGF signaling or endothelial differentiation. Notably, nobiletin prevents new vessel formation without affecting existing vasculature. Other compounds, including rhein, quercetin, berberine, and active constituents of *Euphorbia pekinensis* Rupr. [Euphorbiaceae; *Euphorbiae pekinensis* radix] (e.g., 3,3′-O-dimethoxy ellagic acid), also inhibit angiogenesis through Src/FAK pathway modulation, highlighting the multi-target advantages of natural products in controlling abnormal vascular proliferation (He et al., 2011; Zhao et al., 2014; Zhang W. T. et al., 2022; Nathan et al., 2023).

Some Chinese medicines exhibit bidirectional vascular regulation through distinct active ingredients. *Panax ginseng* C.A.Mey. [Araliaceae; *Ginseng* Radix et Rhizoma] is an example, as its numerous ginsenosides have opposing effects on angiogenesis (Sengupta et al., 2004). Specifically, Rg1, Rb3, and Rc show pro-angiogenic activity in zebrafish models (Leung et al., 2006; Zhu et al., 2023), while Rb1 and Rg3 demonstrate anti-angiogenic activity (Yue et al., 2006; Leung et al., 2007). Processed red ginseng-derived ginsenoside Rh2 (G-Rh2) dose-dependently inhibits intersegmental angiogenesis at concentrations ranging from 30 to 90 μM (Ma et al., 2020). These opposite effects may be associated with structural differences in the sugar chains and hydroxyl substituents among various ginsenosides. In addition, changes in drug dosage may influence their pro-angiogenic and anti-angiogenic activities, exhibiting a characteristic dose-dependent bidirectional regulatory effect (Sengupta et al., 2004). Similarly, *A. sinensis* (Oliv.) Diels [Apiaceae; *Angelicae* sinensis radix]’s volatile component, n-butylidenephthalide, suppresses angiogenesis, contrasting with the pro-angiogenic effects of its aqueous extract (Yeh et al., 2011). These discrepancies highlight the importance of distinguishing extraction methods and compound polarity when interpreting opposite pharmacological outcomes. Naringin from citrus fruits restores vascular loss in zebrafish, whereas its aglycone, naringenin, exhibits anti-angiogenic potential (Chen L. et al., 2018), further illustrating that subtle structural variations can reverse angiogenic activity among natural products.

Alongside the aforementioned individual medicinal components, Chinese herbal compound prescriptions and Chinese patent medicines also exert regulatory influence on angiogenesis. *Erxian Decoction* inhibits angiogenesis in zebrafish by downregulating VEGF-A and HIF-1α (Yu et al., 2012). *Tongnao Decoction* activates the vascular endothelial growth factor receptor-2 (VEGFR-2), phosphoinositide 3-kinase (PI3K), protein kinase B (Akt), and Raf-MEK1/2-ERK1/2 signaling pathways to repair VRI-induced damage to zebrafish ISVs, SIVs, and central arteries (CtAs) (Wang J. L. et al., 2020). Both *Compound Danshen Dripping Pill* (Hu et al., 2022) and *Shuxinyin Formula* (Zhou et al., 2019a) regulate the VEGF/PI3K/Akt/MAPK signaling pathway to enhance angiogenic potential. Additionally, the *Shuxinyin Formula* is also involved in the regulation of cell junctions and autophagy. *Xinkeshu tablets* integrate multiple pathways, including PPAR, AGE-RAGE, and VEGF, to promote angiogenesis (Liu et al., 2023). High dosages of *Danshen Gegen decoction* increased SIVs germination (Hu et al., 2013). Among licorice-derived preparations (such as magnesium isoglycyrrhizic acid injection (MII), diammonium glycyrrhizic acid injection (DGI), compound glycyrrhizic acid tablets (CGT)), DGI exhibits the strongest activity and can increase the length and number of zebrafish ISVs and SIVs in a concentration-dependent manner (Yu et al., 2025). Furthermore, the synergistic effects of salvianolic acid B and ferulic acid in *GXNT tablets* promote angiogenesis by upregulating VEGF receptor expression (Chen et al., 2022), while the active constituents of *Yiqi Fumai Lyophilization Injection* (YQFM) outperform ginsenoside Rb2 (Xu et al., 2023) (Other similar functional research is listed in Table 4)

Zebrafish have unique advantages in studying both pro-angiogenic and anti-angiogenic effects, particularly in investigating these dual effects within the same model system. This capability is of significant importance in cardiovascular disease research, especially in the regulation of angiogenesis, where it holds promise for simultaneously inhibiting abnormal vessel formation while preventing excessive vascular regression. However, many plant metabolites and natural products show biphasic dose-response patterns, which can result in incorrect conclusions if only one concentration is tested (Zhu et al., 2016; Caprifico et al., 2025). Therefore, future studies should carefully examine the dose-response relationships and the timing of angiogenic effects to enhance therapeutic targeting.

### 2.5 Cardiotoxicity model

Zebrafish embryos have been used to screen a variety of toxicants, particularly for cardiotoxicity testing. Table 5 provides a summary of recent studies that use zebrafish to investigate natural product-mediated or -induced cardiotoxicity.

**TABLE 5:** Evaluation of natural products for cardiotoxicity and cardioprotective effects using zebrafish models.

Bioactivity	Source	Extracts/Compounds	Model	Finding	Reference
Reduces cardiotoxicity	*Astragalus membranaceus* (Fisch.) Bunge [Fabaceae; *Astragali* radix]	Calycosin (CA)	Tg (GFP-Lc3) 24hpf zebrafish embryos, adult zebrafish Doxorubicin (DOX) solution (100 μM) for zebrafish embryos One dose of DOX (20 mg/kg) intraperitoneal (IP) injection in adult zebrafish	CA can decrease doxorubicin-induced cardiotoxicity (DIC) in zebrafish embryos, resulting in improved survival, fractional shortening (FS) and heart rate, relieved morphological damage CA can restore the DOX-induced decrease of ejection fraction (EF) in zebrafish CA restored cardiac function and autophagy. nppa ↓,nppb ↓	Lu et al. (2021)
	*Salvia miltiorrhiza* Bunge [Lamiaceae; *Salviae miltiorrhizae* radix et rhizoma]	Salvianolic acid A (SAA)	Wild-type AB strain 24 hpf zebrafish embryos Clozapine (CLZ) (37.5 μmol/L)	SAA can improve the heart edema of zebrafish, increased heart rate, and reduced the SV-BA interval SAA can mitigate clozapine-induced cardiotoxicity. il-1b↓, nfkb2↓, mcl1a↓, mcl1b↓, sod1↑, cat↑	Lin et al. (2022)
Induce cardiotoxicity	*Artemisia annua* L. [Asteraceae; *Artemisiae annuae* herba]	Artesunate (ART)	Wide-type AB strain 2dpf zebrafish embryos Intravenous (IV) inject	MNLC = 32.4ng/fish, LD_10_ = 41.8 ng/fish, LOAEL = 2.5 ng/fish ART has cardioprotective benefits at low dosages, although demonstrates cardiotoxicity at elevated dosages Single intravenous ART (3.6–41.8 ng/fish) caused pericardial edema, dyscirculatory effects, delayed yolk sac absorption, renal edema, and swim bladder loss. fzd7a↓, coro1cb↓, Cdk5rap1↑, Klotho↑, Ptges↑, Slc22a17↑, Creld2↓, Ndufa8↓ and Stat6↓	Zheng et al. (2020)
	*Aesculus hippocastanum* L. [Hippocastanaceae; *Aesculi hippocastani* semen]	Sodium aescinate (SA)	Wide-type AB strain 72 hpf zebrafish larvae Yolk sac microinjection with SA (10 nL each)	MNLC = 1.5 μg/mL,LC_10_ = 2.0 μg/mL Uncovered the potential cardiotoxicity of SA for the first time SA treatment can lead to heart malformations, pericardial edema, and reduced circulation Cardiac and pericardial malformations: SA at 1/10 MNLC and above doses Significant cardiac malfunctions (heart rate and circulation decrease/absence):1/3 MNLC and above doses	Liang et al. (2016)
	*Salvia miltiorrhiza* Bunge [Lamiaceae; *Salviae miltiorrhizae* radix et rhizoma]	Tanshinone IIA (Tan-IIA)	Wild-type Tuebingen strain 2 hpf normal dechorionated embryos	72hpf LC_50_ = 18.5 µM,96 hpf LC_50_ = 12.8 µM Zebrafish embryos treated with different concentrations of Tan-IIA showed pericardial edema, spinal curvature, and tail amputation	Wang et al. (2017)
	*Aconitum carmichaeli* Debx. [Ranunculaceae; *Aconiti lateralis* radix praeparata]	Aconitine, mesaconitine, and hypaconitine	Wide-type AB strain 2 dpf zebrafish embryos	Acute toxicity of the cardiovascular, digestive, developmental, and respiratory systems (including arrhythmia, liver degeneration, yolk sac absorption delay, length decrease, and swim bladder loss): FZ-120 (288–896 μg/mL)	Sun et al. (2018)
	*Momordica cochinchinensis* (Lour.) Spreng [Cucurbitaceae; *Momordicae* fructus]	Cochinchina momordica seed extract (CMSE)	Wild-type AB line and Tg (MPO: GFP) 2dpf to 3 dpf zebrafish larvae	CMSE may elicit cardiotoxicity via mechanisms associated with inflammation, oxidative stress, and apoptosis	Du et al. (2021)
	*Evodia rutaecarpa* (Juss.) Benth. [Rutaceae; *Evodiae* fructus]	Evodiamine	Wild-type AB strain,Tg (cmlc2: EGFP) 72–120 hpf zebrafish larvae	LC_10_= 354 ng/mL, MNLC= 113.4 ng/mL 50–100 ng/mL: no lethal effect, ≥400 ng/mL: sharp increase in lethality,1,600 ng/mL: lethality reached 100% Evodiamine could cause cardiovascular side effects involving oxidative stress	Yang et al. (2017)
	*Aconitum carmichaeli* Debx. [Ranunculaceae; *Aconiti* radix]	Aconitum alkaloids Aconitine (AC) and mesaconitine (MA)	Wild-type AB strain 96hpf zebrafish larvae	96 h-EC_50_ of pericardium edema (Aconitine) = 3.5 μg/L,(Mesaconitine) = 65.7 μg/L 96 h-LC_50_(Aconitine) = 4.2 μg/L,(Mesaconitine) = 72.4 μg/L, 2.5 μg/L AC and 20 μg/L MA resulted in an impaired cardiovascular system, with yolk sac bleeding and early cardiac dysfunction identified at 96 hpf	Liu et al. (2019)
	*Cinnamomum camphora* (L.) J. Presl [Lauraceae; *Borneolum*]	Three isomers of borneol [(−)-borneol, (+)-borneol, and isoborneol]	Wild-type AB strain, Tg (zp3: fsta, myl7: EGFP) 4hpf zebrafish embryos	Three isomers of borneol increased the mortality of zebrafish embryos and decreased their hatching rate. (+) - Borneol has a lower mortality and deformity rate on zebrafish embryos, while isoborneol has a stronger toxicity	Ni et al. (2022)
	*Aconitum carmichaelii* Debx. [Ranunculaceae; *Aconiti lateralis* radix praeparata]	Aconitine	Wild-type AB strain, Tg (cmlc2: eGFP) 48hpf zebrafish embryos	LD_50_= 7.92 μM 2 and 8 μM aconitine decreased the heart rate of embryos significantly, and inhibit the contraction of ventricles and atria	Li et al. (2020b)
	*Anacardium occidentale* L. [Anacardiaceae; *Anacardii* fructus]	Extracts of cashew nutshells	Wild-type AB line ≤3hpf zebrafish embryos	Aqueous extract: Concentration ≥ 62.5 μg/mL: increased cardiac functional area change (FAC), abnormal cardiac contraction Chloroform extract: High toxicity, led to developmental retardation, spinal deformities, yolk sac edema Organic extract: Led to yolk sac edema, spinal deformities	Shinde et al. (2025)
	Zishen Guchong Pill (ZGP)	*Panax ginseng* C. A. Mey. [Araliaceae; *Ginseng* radix et rhizoma], *Cuscuta chinensis* Lam. [Convolvulaceae; *Cuscutae* semen], *Rehmannia glutinosa* (Gaertn.) DC. [Orobanchaceae; *Rehmanniae* radix praeparata], etc. (15 herbs)	Tg (cmcl2: EGFP) 4hpf zebrafish embryos 1 mmol/L IFO as positive control	High doses (>50 μg/mL) caused oxidative stress and cardiac development abnormalities (pericardial edema, decreased heart rate, decrease in the number of RBCs in the heart and a significant increase in the distance between SV and BA; low doses (≤50 μg/mL) had no significant toxicity	Wang et al. (2021)

This summary provides information on the regulatory effects of drugs or natural products on cardiotoxicity, using wild-type and transgenic zebrafish as experimental models. “↑” or “↓” illustrates the regulatory effects of drugs or natural products on different indicators. “↑” indicates increase or upregulation; “↓” indicates reduce or downregulation. dpf: days post-fertilization; hpf: hours post-fertilization. MNLC: maximum nonlethal concentration, LD_10_: lethal dose 10%, LOAEL: lowest observed adverse effect level, EC_50_: median effect concentration, LC_50_: Lethal concentration 50%. SV: sinus vein, BA: arterial bulb.

A study induced cardiotoxicity in adult zebrafish Tg (GFP-Lc3) by injecting doxorubicin (DOX) and subsequently treated the zebrafish with *Astragalus membranaceus* (Fisch.) Bunge [Fabaceae; *Astragali* radix] extract. The results showed that *A. membranaceus* (Fisch.) Bunge [Fabaceae; *Astragali* radix] extract significantly reduced heart damage in zebrafish, improved cardiac function, restored autophagy, and significantly decreased the expression of two pathological markers of cardiac remodeling, natriuretic peptide A (nppa) and natriuretic peptide B (nppB) (Lu et al., 2021). Additionally, salvianolic acid A can counteract clozapine-induced cardiotoxicity, improve zebrafish cardiac edema, increase heart rate, and shorten the SV-BA interval. Salvianolic acid A also downregulates inflammatory response-related genes IL-1b, NFKB2, mCl1a, and mCl1b, and upregulates the expression of SOD1 and CAT (Lin et al., 2022).

In addition to the traditional Chinese medicines mentioned above that can improve cardiotoxicity, numerous studies have shown that some natural products can also induce cardiotoxicity. For instance, experiments involving the microinjection of sodium aescinate (SA) extracted from *Aesculus hippocastanum* L. [Sapindaceae; Aesculi hippocastani semen] into zebrafish yolk sacs have demonstrated that SA treatment can lead to cardiac malformations, pericardial edema, and reduced blood circulation (Liang et al., 2016). Next, zebrafish embryos treated with different concentrations of Tan-IIA exhibited pericardial edema (Wang et al., 2017). In addition, the component FZ-120 of *A. carmichaelii* Debeaux [Ranunculaceae; Aconiti lateralis radix praeparata] radix could induce arrhythmias in zebrafish at a dose range of 288–896 μg/mL (Sun et al., 2018). Components of *A. carmichaelii* Debx. [Ranunculaceae; *Aconiti* lateralis radix praeparata], such as aconitine and meaconitine, may cause defects in the cardiovascular system and significantly reduce the heart rate of zebrafish embryos (Liu et al., 2019; Li M. T. et al., 2020). Moreover, *Momordica cochinchinensis* (Lour.) Spreng. [Cucurbitaceae; *Momordicae cochinchinensis* semen] extract may induce cardiotoxicity by triggering apoptosis, oxidative stress, and inflammatory pathways. Evodiamine may result in oxidative stress-related cardiovascular adverse effects (Yang et al., 2017). High concentrations of cashew nut shell aqueous extract induce cardiac contractile dysfunction in zebrafish embryos. The TCM formulation *Zishen Guchong Pill* exhibits considerable harmful effects on the heart development of zebrafish at elevated concentrations, likely associated with the activation of oxidative stress and apoptotic signaling pathw ays triggered by *Zishen Guchong* Pill (Wang et al., 2021). Furthermore, artesunate (ART), the active substance in *Artemisia annua* L. [Asteraceae; *Artemisiae annuae* herba], has different effects on cardiotoxicity at different doses. Research indicates that when ART is intravenously injected into 2dpf zebrafish larvae, it exhibits cardiotoxicity at high doses but has a cardioprotective effect at low concentrations. This bidirectional regulatory effect may be closely related to the different expression of genes such as Cdk5rap1, Klotho, Ptges, Slc22a17, Creld 2, Ndufa 8 and Stat6 under ART regulation (Zheng et al., 2020).

Zebrafish are an excellent tool for cardiotoxicity screening, capable of rapidly assessing heart rate, contractility, and structural abnormalities. However, their remarkable cardiac regenerative capacity may mask chronic toxicity effects that would emerge in mammals with limited regenerative potential (González-Rosa et al., 2017). Therefore, compounds exhibiting promising or unclear cardiotoxicity profiles in zebrafish should be further evaluated in mammalian models to ensure accurate risk assessment and safety, providing more reliable data for clinical applications.

## 3 Discussion and perspective

Cardiovascular disease has long been a major public health concern, posing a serious threat to human health. Its origins can be traced back to ancient medical practices. Heart-related illnesses are documented in the medical texts of many ancient civilizations. For example, the concepts of the heart and blood vessels appear in ancient Egyptian and Greek medicine (Loukas et al., 2016). Additionally, traditional Chinese medical classics, such as the *Huangdi Neijing* (Yellow Emperor’s Inner Canon), describe symptoms like “palpitations” and “chest impediment,” attributing their causes primarily to visceral dysfunction and disturbances in the flow of Qi and blood. Therefore, in traditional therapies, the primary function of natural products in improving CVDs is to promote blood circulation and remove blood stasis. Furthermore, the function of the heart is closely linked to the circulation of Qi and blood in the body, making Qi-tonifying Chinese medicines commonly used in the treatment of CVDs (Li D. X. et al., 2024; Si and Yu, 2024). Here, “Qi” can be understood as a vital life energy or vitality that helps the body’s various parts function properly. Additionally, traditional medicines are used to eliminate excess heat and toxins from the body. These medicines are commonly applied to treat cardiovascular issues caused by inflammation, internal heat, or harmful substances (Wang et al., 2022). In modern pharmacology, these natural products also demonstrate effects in regulating blood lipids and lowering blood pressure (Li Y. P. et al., 2017).

The application of natural products in the treatment of CVDs has been confirmed by modern pharmacological studies. For example, *S. miltiorrhiza Bunge [*Lamiaceae*; Salviae miltiorrhizae* radix et rhizoma] and *P. notoginseng* (Burkill) F.H.Chen [Araliaceae; *Notoginseng* radix et rhizoma] are commonly used to promote blood circulation and prevent thrombosis. Studies have shown that their active ingredients, such as tanshinone, salvianolic acid B, notoginsenoside R1, Rg1, and Rb1, exert multi-target protective effects. These compounds improve blood circulation, exhibit anti-thrombotic activity, and demonstrate therapeutic effects on conditions such as HF and other CVDs (Yin et al., 2020; Ma et al., 2021). As a representative herbal medicine for nourishing qi and nourishing the heart, ginseng plays its role by enhancing heart function and restoring the flow of qi in the body. Modern studies have found that ginsenosides can improve cardiac blood flow and heart rate, and when used in combination with *Aconitum carmichaeli Debx.* [Ranunculaceae*; Aconiti lateralis* radix praeparata], they enhance cardiac output (Li et al., 2025). *Scutellaria baicalensis Georgi* [Lamiaceae*; Scutellariae* radix] and *C. longa* L. [Zingiberaceae; *Curcumae* longae rhizoma] are widely used in the treatment of CVDs due to their heat-clearing, detoxifying, lipid-lowering, and anti-inflammatory effects (Jin et al., 2011; Seo et al., 2014; Zhang et al., 2025). In addition, *Senna tora* (L.) Roxb. [Fabaceae; *Semen cassiae* torae] (Juemingzi), traditionally known for its heat-clearing and detoxifying properties, has also shown significant lipid-lowering effects in zebrafish studies, particularly in reducing plasma cholesterol levels (Zhou C. et al., 2024). By combining traditional medicine with modern pharmacological research, the zebrafish model has become an effective tool to validate the therapeutic mechanisms of natural products, further advancing the modernization of traditional Chinese medicine in the treatment of CVDs.

The analysis of various natural products reveals that they have a certain inherent consistency in the regulation of cardiovascular function. Although these active compounds are derived from different plant sources, they often act in similar ways to exert cardiovascular benefits. Studies have shown that many natural compounds regulate common key signaling pathways—especially by activating the VEGF/PI3K/Akt pathway, reducing oxidative stress and inhibiting inflammation-thereby promoting blood vessel repair, improving endothelial function and enhancing cardiomyocyte survival (Liu et al., 2021; Zhong et al., 2024). This convergence of mechanisms reflects the coordinated regulation of the signal network by natural products at the system level.

Based on the above understanding, this article reviews the combination of zebrafish cardiovascular disease model and natural product research, focusing on thrombosis, angiogenesis, HF, hyperlipidemia and cardiotoxicity and other conditions. In thrombosis studies (Table 1), most experiments use arachidonic acid-induced models, and the concentration of natural products is usually 1–100 µg/mL. It is worth noting that TCM formulas usually show pharmacological activity at higher concentrations than a single compound, which indicates that there may be synergistic effects between multiple ingredients. In hyperlipidemia studies (Table 2), models rich in cholesterol and egg yolk feeding are the most common. Natural products generally reduce cholesterol and triglyceride levels by about 30%–50%, which is confirmed by oily red O staining of vascular lipid deposits. Comparative analyses identified several multitarget compounds—such as salvianolic acid B and ginsenosides—with antithrombotic, cardioprotective, and proangiogenic effects (Qi et al., 2017; Chen et al., 2022; Ma et al., 2021; Dong et al., 2022), indicating that single-pathway evaluations may underestimate their therapeutic potential. Methodologically, most studies relied heavily on larvae 48–72 h post-fertilization, suggesting future work could explore adult zebrafish models or different developmental stages.

Compared to traditional animal models, zebrafish offer advantages such as high reproductive capacity, low cost, transparent embryos, and ease of real-time imaging, making them ideal for high-throughput drug screening and genetic research. With their genome highly homologous to that of humans, combined with advanced gene-editing techniques, zebrafish provide unique conditions for functional gene validation and the study of drug mechanisms. From an ethical perspective, zebrafish are increasingly preferred over rodent models because of their reduced sentience, simpler nervous system, and lower ethical burden in experimental use. Their use also aligns with the 3Rs principles (Replacement, Reduction, and Refinement), promoting more ethical and efficient research. These advantages make zebrafish an attractive option for early-stage pharmacological studies, particularly in natural product screening (Cassar et al., 2020). However, zebrafish have limitations for clinical translation: their heart has only one atrium and ventricle, lacking mammalian pulmonary circulation, and drugs are absorbed passively through water, unlike human administration. Zebrafish have only one atrium and one ventricle, which changes the pressure and flow pattern of the blood, resulting in the mixing of oxygenated and deoxygenated blood. Despite this, zebrafish and mammals still have high similarities in cardiac contraction, electrical conduction, and many basic signaling pathways (Genge et al., 2016; Nguyen et al., 2008). Because zebrafish do not have pulmonary circulation, they cannot simulate diseases such as pulmonary hypertension or right heart failure, which are important parts of human cardiovascular disease. Moreover, we emphasize that zebrafish are not a simple replacement for mammalian models, but rather a research system with complementary advantages, playing a unique role in high-throughput screening, real-time imaging, and complex natural product studies. However, mammalian models are closer to humans in terms of anatomy, physiology, and pharmacokinetics (Chahardehi et al., 2020). Therefore, the zebrafish model is more suitable for early-stage drug efficacy screening and mechanism studies, while preclinical validation still requires the use of mammalian models. Combining these two approaches enables more comprehensive and efficient cardiovascular research.

Zebrafish cardiovascular studies show notable methodological differences, particularly in the choice of strain, dosing strategies, and measurement approaches. The AB and TU wild-type strains are most commonly used for general toxicity and efficacy testing (van de Venter et al., 2020), while transgenic lines such as Tg (fli1:EGFP) and Tg (myl7:DsRed) provide valuable imaging advantages (Choe et al., 2021). However, these transgenic models may display physiological differences from wild-type fish, making it important for researchers to clearly report strain-specific details. Inconsistent dose selection, often without pharmacokinetic justification or complete dose–response analysis, remains a key limitation (Cortemeglia and Beitinger, 2005; Holden and Brown, 2018). Future research should aim to quantify exposure levels, ensure chemical stability, and conduct negative controls. Differences in endpoints such as red blood cell counts, pericardial area, and blood flow velocity further impact comparability. Moreover, reproducibility can be affected by environmental and biological factors, including temperature, circadian rhythm, developmental stage, and clutch-to-clutch variability. Addressing these issues through standardized experimental protocols and transparent reporting will greatly enhance the reliability and translational relevance of zebrafish-based cardiovascular research.

It is worth noting that the excellent heart regeneration ability of zebrafish represents an innovative tool for natural product research. However, there are still challenges in TCM research, especially the underutilization of this regenerative ability. As an ideal model for heart regeneration research, zebrafish has developed a series of advanced technologies that provide strong support for mechanical analysis. For example, tools such as gene editing enabling precise knockout or overexpression of regeneration-related genes), optogenetics (which regulates cardiomyocyte proliferation) (Baillie et al., 2021), and single-cell sequencing (enabling the analysis of regenerative responses in cardiomyocyte subsets) are instrumental in supporting mechanistic studies (Luz et al., 2025). Despite the establishment of cardiac regeneration models in developmental biology for drug screening, literature over the past decade shows minimal application of these models in TCM or natural product research. While modern medicine uses zebrafish models to elucidate key pathways (e.g., the VEGFC-EMILIN2A-CXCL8A axis) (El-Sammak et al., 2022), TCM research still relies on traditional indicators, such as heart rate and pericardial edema, failing to fully leverage advanced technologies to uncover the mechanisms of natural products.

In addition, the application of the zebrafish model has extended to multiple fields, including inflammation, injury repair, and regeneration. For example, in the *Dextran sulfate sodium salt* (DSS)-induced zebrafish inflammatory bowel disease (IBD) model, *Chrysanthemum morifolium* Ramat. [Asteraceae; *Chrysanthemi caulis* et folium] extract exerts anti-inflammatory and antioxidant effects by inhibiting inflammatory factors such as IL-1. In a spinal cord injury model, feeding fish food containing fig extract accelerates swimming function recovery and promotes spinal cord regeneration (Motohashi et al., 2025). In the caudal fin regeneration model, *Rehmannia glutinosa* (Gaertn.) DC. [Orobanchaceae; *Rehmanniae* radix praeparata] extract significantly promotes tail fin regeneration (Chen et al., 2024). These studies have shown that zebrafish not only has important value in cardiovascular pharmacology, but also provides strong support for the application of natural products in other disease fields. Since natural product extracts often exhibit overall regulatory effects, it is difficult to fully reflect their pharmacological activity with a single indicator. Integrating multiple omics techniques, such as transcriptomics and metabolomics, can help identify key regulatory genes. For example, combined transcriptomic-metabolomic analysis has demonstrated how *Achillea alpina* L. [Asteraceae; Achilleae herba] essential oil modulates amino acid metabolism and inflammatory pathways to exert anti-inflammatory effects (Cheng et al., 2025). This approach can advance cardiovascular research by enabling a systematic analysis of the multi-target molecular networks in TCM and natural products, revealing their connections to key nodes in cardiovascular pathophysiology. Integrating zebrafish’s heart regeneration abilities with natural product research can uncover potential molecular targets in TCM formulas or compounds and deepen the understanding of their therapeutic effects. With the development of multi-omics and real-time imaging technologies, zebrafish are poised to become a key platform for cardiac regeneration research in TCM, aiding in the discovery of new therapeutic strategies and drug candidates. Future studies should combine multi-omics data with zebrafish high-throughput screening to create a “component-target-pathway” framework and explore natural products’ unique regulatory mechanisms in heart regeneration.

In short, natural products have shown strong potential in the treatment of CVDs, including managing blood lipids, promoting angiogenesis, and fighting atherosclerosis and HF, usually through multi-target mechanisms. The zebrafish model has the advantages of high-throughput screening and similar to human disease pathways. It is a key tool for exploring its efficacy and mechanism, and provides new insights for the prevention, treatment and drug development of CVDs.

## Glossary

AA: arachidonic acid
ACC1: acetyl-CoA carboxylase 1
BA: arterial bulb
BAX: BCL2-associated X protein
Bcl-2: B-cell lymphoma-2
CD36: cluster of differentiation 36
CO: cardiac output
COX/cox: cyclooxygenase
CPT1: carnitine palmityl transferase Ⅰ
CtAs: central arteries
CV: caudal vein
CVDs: cardiovascular diseases
DA: dorsal aorta
DLAV: dorsal longitudinal anastomotic artery
DOX: doxorubicin
Dpf: days post-fertilization
**EC**_**50**_: median effect concentration
EF: ejection fraction
F7: coagulation factor VII
FAC: functional area change
FGA/fga: fibrinogen α chain gene
FGB/fgb: fibrinogen β chain
FII: coagulation factor II
FIX: coagulation factor IX
FS: fractional shortening
FVII: coagulation factor VII
FX: coagulation factor X
GPⅡb/Ⅲa: glycoprotein IIb/IIIa
HDL: high-density lipoprotein
HF: heart failure
hpf: hours post-fertilization
IL: interleukin
ISVs: intersegmental vessels
JNK: c-Jun N-terminal kinase
Kdrl: kinase insert domain receptor like
**LC**_**50**_: lethal concentration 50%
**LD**_**10**_: lethal dose 10%
LDL: low-density lipoprotein
LOAEL: lowest observed adverse effect level
MARK: mitogen-activated protein kinase
MDA: malondialdehyde
mmp9: matrix metalloprotein 9
MNLC: maximum nonlethal concentration
mTOR: mammalian target of rapamycin
NF-κB: nuclear factor kappa-B
NOS3: nitric oxide synthase 3
Notch1a: notch receptor 1a
nppA: natriuretic peptide A
nppB: natriuretic peptide B
OAs: optic arteries
PHZ: phenylhydrazine
PI3K-Akt: phosphatidylinositol-3-kinase/protein kinase B signaling pathway
PPAR: peroxisome proliferator-activated receptor
ptgs2b: prostaglandin-endoperoxide synthase 2 variant B
RBCs: red blood cells
SIVs: subintestinal vessels
SOD: superoxide dismutase
Src-FAK: steroid receptor coactivator/focal adhesion kinase pathway
SREBP: sterol-regulatory element binding proteins
Stat6: signal transducer and the activator of the transcription 6
SV: sinus vein
TC: total cholesterol
TCM: traditional Chinese medicine
TF: coagulation factor III
TG: triglyceride
TNF-α: tumour necrosis factor-alpha
TXA2: thromboxane A2
UCP2: uncoupling protein 2
VEGF: vascular endothelial growth factor
VLDL: very low-density lipoprotein
VRI: VEGF receptor tyrosine kinase inhibitor II
